# Epidemiological overview of multidimensional chromosomal and genome toxicity of cannabis exposure in congenital anomalies and cancer development

**DOI:** 10.1038/s41598-021-93411-5

**Published:** 2021-07-06

**Authors:** Albert Stuart Reece, Gary Kenneth Hulse

**Affiliations:** 1 Division of Psychiatry, University of Western Australia, Crawley, WA 6009 Australia; 2 School of Medical and Health Sciences, Edith Cowan University, Joondalup, WA 6027 Australia

**Keywords:** Cancer, Developmental biology, Ecology, Genetics, Environmental sciences, Environmental social sciences, Diseases, Medical research, Oncology, Pathogenesis, Risk factors

## Abstract

Cannabis and cannabinoids are implicated in multiple genotoxic, epigenotoxic and chromosomal-toxic mechanisms and interact with several morphogenic pathways, likely underpinning previous reports of links between cannabis and congenital anomalies and heritable tumours. However the effects of cannabinoid genotoxicity have not been assessed on whole populations and formal consideration of effects as a broadly acting genotoxin remain unexplored. Our study addressed these knowledge gaps in USA datasets. Cancer data from CDC, drug exposure data from National Survey of Drug Use and Health 2003–2017 and congenital anomaly data from National Birth Defects Prevention Network were used. We show that cannabis, THC cannabigerol and cannabichromene exposure fulfill causal criteria towards first Principal Components of both: (A) Down syndrome, Trisomies 18 and 13, Turner syndrome, Deletion 22q11.2, and (B) thyroid, liver, breast and pancreatic cancers and acute myeloid leukaemia, have mostly medium to large effect sizes, are robust to adjustment for ethnicity, other drugs and income in inverse probability-weighted models, show prominent non-linear effects, have 55/56 e-Values > 1.25, and are exacerbated by cannabis liberalization (P = 9.67 × 10^–43^, 2.66 × 10^–15^). The results confirm experimental studies showing that cannabinoids are an important cause of community-wide genotoxicity impacting both birth defect and cancer epidemiology at the chromosomal hundred-megabase level.

## Introduction

Cannabinoid-induced genotoxicity was first demonstrated by researchers in the 1960’s who showed multiple congenital defects developing in prenatally exposed animals^1–3^, cannabinoid-induced micronucleus formation from chromosomal mis-segregation errors and mitotic spindle disruption^4^, ring and chain chromosomal malformations in sperm^5^, nuclear blebbing and bridging of oocytes and lymphocytes during cytokinesis^6,7^ and direct and indirect multimodal mitochondrial toxicities with downstream direct and indirect epigenetic effects^4,8–12^. It has long been known that cannabinoids reduce histone formation and protamine substitution and synthesis resulting in a more open chromosomal conformation which is more subject to mutagenicity and is also pro-oncogenic as more genes are available for transcription^13–18^. Cannabis has a large epigenetic footprint with major alterations of DNA methylation, a change inheritable to subsequent generations in both mice and man^9,19–26^.

An elegant and incisive molecular dissection of the cannabidiol-related genotoxic mechanisms was recently published by the Parnell group which indicated that cannabidiol-hedgehog signalling and cannabinoid receptor type 1 (CB1R)—smoothened receptor heterodimerization was a key molecular mediator of developmental malformations including orofacial cleft palate and lip deformities, exencephaly and microphthalmia/anophthalmia in mice and zebrafish^27^. These authors also noted that since the hedgehog pathway is a key developmental mechanism also implicated in many oncogenic pathways it could also be expected to implicated in the growth and promotion of several cancers. This important predictive hypothesis has not been tested epidemiologically to our knowledge. If confirmed it would clearly carry major public health and regulatory implications as it seems very evident that the genotoxicity of many cannabinoids is not well appreciated currently in either lay or professional circles. Moreover promiscuous heterodimerization of the CB1R with many other G-protein receptors has been reported including notch and CBR2^28^, delta^29^ and mu^30^ opioid receptors, angiotensin II^31^, serotonin 2A receptors^32^, GPR55^33^, orexin^34^, dopamine type 2A^35^ and adenosine 2A^36^. Heterodimerization of the CB1R changes its downstream interactions with G-protein transduction coupling machinery and can change the polarity of signalling from inhibition to activation^27^.

Presumptive evidence for cannabinoid-induced genotoxicity was first demonstrated in human populations in Hawaii with the 2007 documentation of elevated rates of Down syndrome in infants prenatally exposed to cannabis but not to other drugs, with OR 5.26 (95% CI 1.08–15.46)^37^. This finding has since been confirmed in Colorado and Australia^8,38^ and by studies of US data^39^. Indeed a dramatic rise from the fourth to the fifth quintiles of cannabis use amongst US states was recently reported for 34 congenital anomalies of birth including prominently cardiovascular and central nervous system disorders, orofacial clefts, limb reductions and the chromosomal disorders Down syndrome, Trisomy 13, Turner syndrome and Deletion 22q11.2^39^.

Testicular cancer, with its ubiquitous chromosome 12 anomalies^40–42^, has been linked with parental cannabis exposure in all four studies to examine this relationship^43–46^.

Together this list makes an impressive assemblage of the human genome. Chromosomes 12, 13, 18, 21, and X are each 133, 114, 80, 48 and 153 megabases (MB) in length so together they comprise 528 MB (16.7%) of the human genome which is in total about 3000 MB in length. This substantial list also leaves open the possibility that cannabis may be generally toxic to human chromosomes including the possibility that damage related to other chromosomes is filtered out by in utero foetal loss contributing to the lower fecundity and higher miscarriage rates known to occur amongst human women who consume cannabis^47,48^.

Cannabis use in parents has previously been linked with non-lymphoblastic leukaemia, several pediatric sarcomas^49–51^ and in a recent causal inference report, with the rising rate of pediatric cancers across USA since 1970^52^. This latter is important as it presents presumptive clinical evidence of intergenerational inheritance of oncogenic mutagenicity and teratogenicity^53,54^.

Whilst the relationships between cannabinoids and various morbidities are increasingly being studied, it seemed timely to review the epidemiological evidence linking cannabinoid-induced genotoxicity to clinical phenomenology at the epidemiological level using US space–time denominated drug and disorder data which is the perhaps the most complete dataset globally using standard epidemiological tools. The objective here is to provide in overview form an increased understanding of the implications of cannabinoid induced genotoxicity, areas not well understood by medical or public health practitioners or in the general community.

Principal Components (PC) analysis is a classical statistical technique which quantifies the dominant trends in a cloud of data points and allows several variables to be combined at once thereby allowing significant dimension reduction and streamlining of data analysis across multiple variables.

The central hypothesis to be investigated was whether there was a relationship between cannabis use and Principal Components (PC’s) of the congenital anomalies Down syndrome, Trisomy 18 (Edwards syndrome), Trisomy 13 (Patau’s syndrome), Turner syndrome, Deletion 22q11.2 (Di George syndrome), and selected cancers namely thyroid, liver, breast and pancreatic cancers and acute myeloid leukaemia, and whether the association fulfilled formal quantitative criteria of causality. The present study was intended to be an overview and introduction to the potentially broad impacts of cannabinoid genotoxicity across the chromosomal complement and was useful to introduce the concept of large-scale genetic damage. Rather than considering morbidities separately it was felt that additional insights could be gained by considering these syndromes together particularly with regard to their impacts across the chromosomal landscape. This is not of course to suggest that detailed studies on each pathology mentioned should not be conducted. And indeed for many of these issues we are doing just this at the time of writing. However it was felt that much could be gained by the “wide-angled lens approach” in parallel with detailed spatiotemporal and causal inference epidemiological analyses.

Our concerns in relation to cannabis and cannabinoids were heightened by the recent demonstration from SAMHSA that cannabis use alone has risen across this period whilst the use of tobacco and alcohol use disorder have declined^55–57^ and by the demonstration that the rate of daily or near daily cannabis consumption in USA has recently doubled again based on SAMHSA data^58^. The rates of opioid use in household surveys has generally declined and the rate of cocaine use has been consistently low level.

The emerging picture is in fact very concerning and indeed the very antithesis of the supposedly “soft drug image” with which cannabis is invariably associated in popular culture. Epidemiological data implicates several cannabinoids including cannabigerol, cannabinol and cannabichromene in addition to tetrahydrocannabinol (THC). Public health concerns are heightened by the major theme coming through much cannabinoid genotoxicity and cell biology of an exponential dose–response relationship^14,26,59–61^ which appears to have serious impacts in environments where cannabis use is allowed to increase—with major multigenerational and transgenerational implications.

## Results

Cancer data was downloaded for the fifty US states from 2001 to 2017 from the SEER registry^62^. Congenital birth anomaly data was taken from NBDPN CDC annual reports^63^. It was adjusted to include estimates of early termination of pregnancy for anomaly (ETOPFA) taken from the published literature^64–66^. These datasets were matched with drug use data from NSDUH at SAMHSA for the period 2003–2017^67^ so that the fifteen years 2003–2017 became the period of analysis. There were therefore 750 datapoints for analysis. This data was supplemented by income and ethnicity data from US census bureau and cannabinoid concentration data from DEA^68–70^.

Cannabis use quintiles were calculated for each year. The mean percentage rates of cannabis use are shown in Supplementary Table 1.

Four Principal Components (PC’s) were constructed in the domains of congenital anomaly (CA) rates (CAR), ETOPFACAR’s, cannabinoid exposure and the five cancers of interest. Details of the composition of the PC’s are shown in Supplementary Tables 2–5.

**Figure 1:**
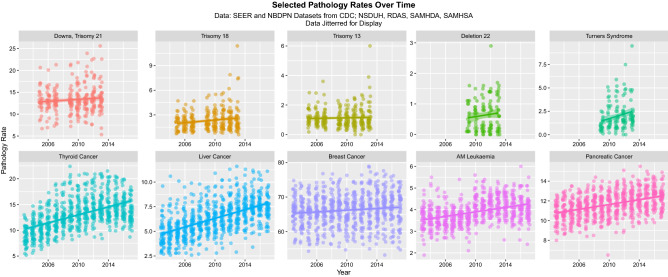
Raw congenital anomaly and cancer incidence data over time, by pathology type. (Created in R-Studio version 1.3.1093 using ggplot version 3.3.2).

**Figure 2:**
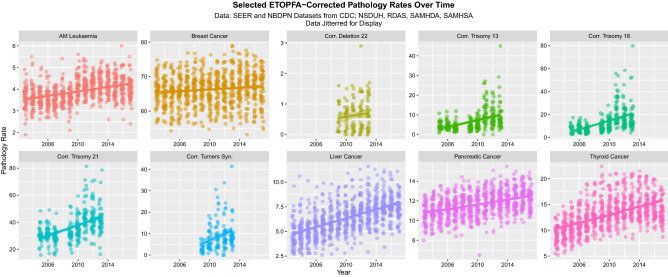
ETOPFA-corrected congenital anomaly and cancer incidence data as a function of THC exposure, by pathology type. (Created in R-Studio version 1.3.1093 using ggplot2 version 3.3.2).

Figures 1 and 2 show the rate of the various disorders over time for raw data and after ETOPFA-adjustment respectively. In all cases the dependent variables listed were rising with time (Fig. 1). ETOPFA adjustment for the congenital anomalies has the effect of exacerbating this increase (Fig. 2). Note the change in scale between graphs.

**Figure 3:**
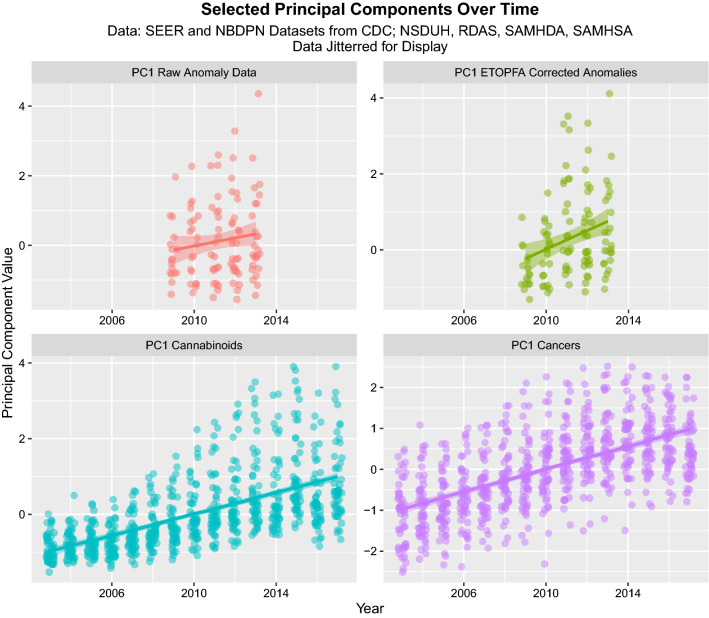
The time course of the four PC1’s for: CAR, ETOPFACAR, cannabinoids and Cancer data (Created in R-Studio version 1.3.1093 using ggplot2 version 3.3.2).

Figure 3 shows the time course of four first Principal Components 1. In each case they are also noted to be rising strongly across time.

**Figure 4:**
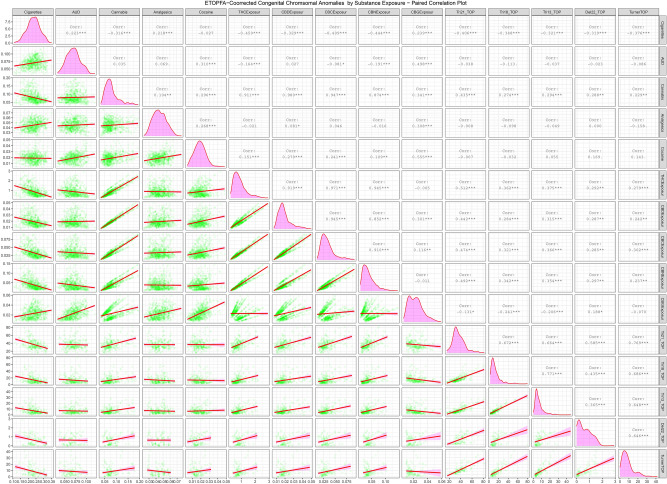
Paired scatterplot matrix for the covariates in the ETOPFACAR dataset. (Created in R-Studio version 1.3.1093 using ggpairs function from the GGally package version 2.0.0).

**Figure 5:**
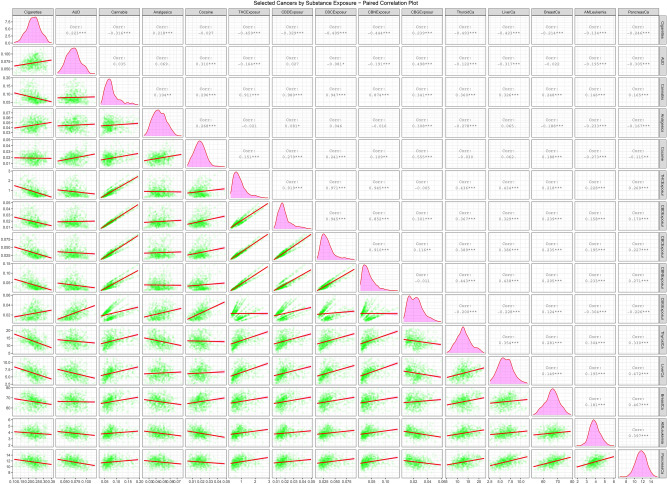
Paired scatterplot matrix for the covariates in the cancer dataset. (Created in R-Studio version 1.3.1093 using ggpairs function from the GGally package version 2.0.0).

Supplementary Fig. 1 and Figs. 4 and 5 are paired scatterplot matrices showing the bivariate relationship of the raw CAR’s, ETOPFACAR’s and cancer rates with substance exposures respectively. In Supplementary Fig. 1 the five chromosomal anomalies appear in the last five rows of the plot matrix and the cannabinoid exposures appear in the middle columns. Cannabis exposure appears in the third column from the left. The relationship between exposures to cannabis and cannabinoids is therefore seen in the positive slopes of the regression lines in the intersection of these rows and columns. Figure 4 is very similar to this but uses the ETOPFA-adjusted data. In this scatterplot matrix it is noted that the slopes of the regression lines in the corresponding plots is much more steeply positive. The relationship of these CAR’s with cannabigerol is different in this plot matrix as most anomalies have a negative relationship with cannabigerol exposure.

Figure 5 performs a similar function for the cancer data. In this plot matrix the cancers occupy the last five rows. The slope of the regression lines for cannabis and the cannabinoids THC, cannabidiol, cannabichromene, and cannabinol is strongly positive. In the case of cannabigerol the relationship is more heterogeneous.

**Figure 6:**
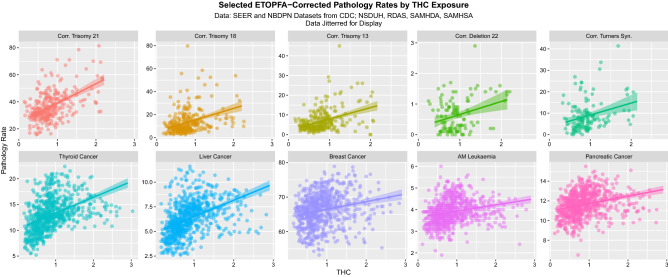
Paired scatterplot matrix for the covariates in the cancer dataset. (Created in R-Studio version 1.3.1093 using ggplot2 version 3.3.2).

Supplementary Figs. 2–6 and 7–10 and Fig. 6 show the relationship between the pathologies of interest and the various cannabinoids in more detail for CAR’s, ETOPFACAR’s and cancers respectively. These figures make explicit the positive relationship between CAR’s and cancers and cannabis, THC, cannabinol, and cannabichromene (Supplementary Figs. 2–5) which is increased by ETOPFA correction (Fig. 6 and Supplementary Figs. 7–9) whilst the relationship between these pathologies and cannabigerol is more complex (Supplementary Figs. 6 and 10).

**Figure 7:**
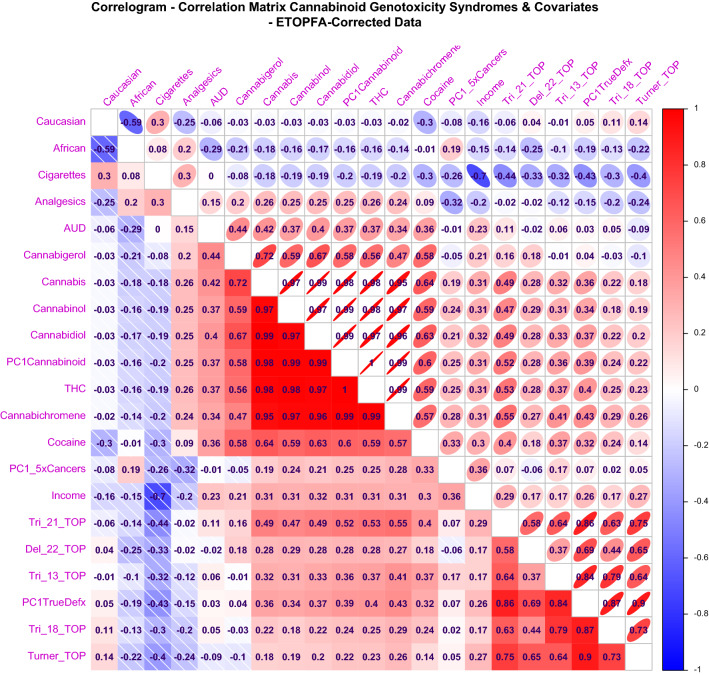
Correlogram drawn in Corrplot for the Pearson correlation coefficients between covariates for ETOPFA-corrected data. The colouring is scaled from strongly positive (bright red) to strongly negative (bright royal blue). The upper triangle represents these associations as ellipses where the width of the ellipses in inversely proportional to the strength of the correlation so that the strongest associations have the narrowest ellipses. Ellipses slope to the right for positive relationships and to the left for negative relationships. (Created in R-Studio version 1.3.1093 using corrplot version 0.84).

**Figure 8:**
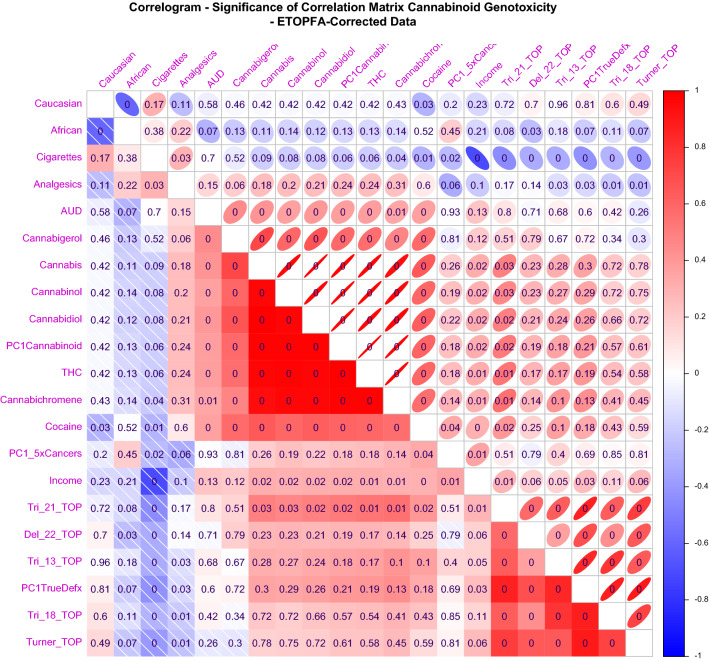
Correlogram drawn in Corrplot for the significance of correlative relationships between covariates for ETOPFA-corrected data. Numbers represent the P-values corresponding to the Pearson correlation coefficients. The colouring is scaled from strongly positive (bright red) to strongly negative (bright royal blue). The upper triangle represents these associations as ellipses where the width of the ellipses in inversely proportional to the strength of the correlation so that the strongest associations have the narrowest ellipses. Ellipses slope to the right for positive relationships and to the left for negative relationships. (Created in R-Studio version 1.3.1093 using corrplot version 0.84).

Supplementary Figs. 11 and 12 and Figs. 7 and 8 show Corrplot correlograms of the correlation coefficients and their significance for the CAR’s and ETOPFACAR’s and cancers respectively. In each case the congenital anomalies appear in the right hand columns along with their combined principal component which is PC!DefxRaw in Supplementary Figs. 11 and 12 and PC1TrueDefx in Figs. 7 and 8. All four correlograms include the PC1Cannabinoid for cannabinoids and PC1_5xCancers which combines the data for the five cancers. In each case the cannabinoids occupy the middle rows.

Positive Pearson correlation coefficients are noted for the CAR’s (Supplementary Fig. 11) which are shown in Supplementary Fig. 12 to be significant for Down syndrome but not for other CAR’s. ETOPFA adjustment has little effect on these correlation coefficients (Fig. 7) and does not change the significance levels of the correlations appreciably (Fig. 8).

Supplementary Figs. 13–15 present time-based cannabis use quintile plots and boxplots for CAR’s, ETOPFACAR’s and cancers respectively. One reads the boxplots by noting where the notches do not overlap which signifies statistically significant differences. The cannabis use quintile categories may be dichotomized as the highest quintile against the lower four quintiles. In each case time-dependent dichotomization reveals very different trends for the highest and lower quintiles (Supplementary Figs. 13–15, panels C and D).

The statistics applicable to the continuous quintile data in these three domains are shown in Supplementary Table 6. These results confirm formally the visual impressions from inspection of Supplementary Figs. 13–15 of important changes at higher quintiles of cannabinoid exposure with high levels of statistical significance (CAR’s: β-est. = 1.76 (1.22, 2.29), P = 3.81 × 10^–9^; ETOPFACAR’s: β-est. = 1.59 (1.04, 2.14), P = 1.15 × 10^–7^; cancers: β-est. = 0.43 (0.26, 0.61), P = 1.69 × 10^–6^).

The statistics for the categorical quintile data confirm the visual impressions of no significant differences between quintiles on the Chi Squared test for trend (ChiSqu. = 444, 444, 3000, df = 440, 440, 2996, P = 0.44, 0.44, 0.48 respectively) but statistically significant differences on comparison of dichotomized data on testing for Student’s T (CAR’s: t = 5.221, df = 16.583, P = 7.49 × 10^–5^; ETOPFACAR’s: t = 4.296, df = 16.08, P = 5.49 × 10^–4^, cancers: t = 5.17, df = 251.52, P = 4.76 × 10^–7^).

Table 1 shows Cohen’s D which is a measure of effect size, its qualitative meaning, and applicable E-Values and P-Values for various quintile dichotomizations. The effect sizes are noted to be large for the CAR’s and ETOPFACAR’s for all comparisons and medium for the cancer comparisons between the third and fifth quintiles.

**Table 1:** Quintile analysis.

	Quintiles 3 v 5	Quintiles 4 v 5	Quintile 5 v lower
**Cohen's D**			
PC1 defects	2.51 (1.61, 3.42)	1.85 (1.05, 2.65)	1.78 (1.18, 2.37)
PC1 ETOPFA	2.09 (1.25, 2.93)	1.53 (0.77, 2.29)	1.57 (0.98, 2.16)
PC1 cancers	0.54 (0.23, 0.85)	0.16 (0.14, 0.47)	0.44 (0.26, 0.62)
**Effect sizes**			
PC1 defects	Large	Large	Large
PC1 ETOPFA	Large	Large	Large
PC1 cancers	Medium	Negligible	Small
**E-Values**			
PC1 defects	19.25, 11.25	10.21, 6.24	9.57, 6.652
PC1 ETOPFA	12.90, 7.75	7.52, 4.64	7.86, 5.43
PC1 cancers	2.91, 2.63	1.62, 1.38	2.68, 2.52
**P-levels of T-tests**			
PC1 Defects	2.81E-06	4.13E-05	7.49E-05
PC1 ETOPFA	3.43E-05	4.45E-04	5.49E-04
PC1 Cancers	4.41E-04	0.2777	4.72E-06

Cohen’s D, Effect Sizes, E-Values and P-values for quintile analyses.

Supplementary Tables 7–9 show the results of inverse probability-weighted instrumental variable regression against PC1 across the three domains in increasingly complex models. The first model shown in Supplementary Table 7 is a model additive for drugs. It is followed by a model interactive for drugs. The third and fourth models are again additive and interactive respectively but substitute the cannabinoids THC, cannabigerol and cannabichromene in place of cannabis. The fifth and sixth models are additive and interactive respectively and include the PC1 of cannabinoids with all the covariates including six ethnicities and median household income. All six models listed include cannabinoids with highly significant positive terms (from β-est. = 3.67 (2.77, 4.56), P = 3.06 × 10^–12^) in model four) and in all six models the effect of cannabinoids is positive overall.

Supplementary Table 8 is structured similarly to Supplementary Table 7 but uses the ETOPFA-adjusted data and includes an additive and an interactive comprehensive model with all covariates including ethnicity and income and the three cannabinoids THC, cannabigerol and cannabichromene. Seven of the eight models listed in this Table incorporate positive and highly significant terms including cannabinoids (from β-est. = 5.40 (3.79, 7.00), P = 2.16 × 10^–9^ in model six) and in six models the overall effect of cannabinoids is positive (models six and eight being the exceptions).

Supplementary Table 9 is structured similarly to Supplementary Table 8 and in this Table the dependent variable is the PC1 for the cancers. All eight final models include terms positive and significant for cannabinoids (from β-est. = 14.84 (10.88, 18.79), P < 2.20 × 10^–16^ in model three; β-est. = 0.35 (0.29, 0.41), P < 2.20 × 10^–16^ in model five; β-est. = 1.48 (1.19, 1.77), P < 2.20 × 10^–16^ in model seven) and in five models the overall effect of cannabinoids is positive (models two, four and eight being the exceptions).

Thus in all three instrumental variable regression tables Supplementary Tables 7–9 terms for cannabis, cannabinoids and PC1-cannabinoids are noted to be significant and positive.

Tables 2, 3, and 4 perform a similar function for inverse probability-weighted robust generalized regression models across the three domains. Table 2 presents final inverse probability weighted robust generalized linear regression models for the CAR dataset. Additive and interactive models for drugs, additive and interactive models for drugs including cannabinoids and additive and comprehensive models including sociodemographic and socioeconomic covariates are presented. Terms including cannabinoids are positive and highly significant in five of the six final models shown and in the first, third, fourth and fifth models the effect of rising cannabinoid exposure is positive overall.

**Table 2:** Robust IPW-weighted generalized regression analyses on PC1 for raw congenital anomaly data.

Parameter	Estimate (C.I.)	P-Value
**Drugs—additive model**		
svyglm(PC1 raw anomalies ~ cigarettes + AUD + mrjmon + Analgesics + cocaine + MHY)		
Cannabis	1.56 (0.4, 2.72)	0.0071
Cigarettes	− 14.87 (− 19.87, − 9.87)	3.83E−06
**Drugs—interactive model**		
svyglm(PC1 raw anomalies ~ cigarettes * AUD * mrjmon + analgesics + cocaine + MHY)		
AUD	74.69 (46.65, 102.74)	1.88E−05
Cigarettes: AUD	− 289.8 (− 428.68, − 150.92)	3.70E−04
**Cannabinoids—additive model**		
svyglm(PC1 raw anomalies ~ cigarettes + AUD + THC + CBG + CBC + analgesics + cocaine)		
CBG	1.42 (0.3, 2.55)	0.0203
Cigarettes	− 14.85 (− 19.8, − 9.89)	3.41E−06
**Cannabinoids—interactive model**		
svyglm(PC1 raw anomalies ~ cigarettes * AUD * THC * CBG * CBC + Analgesics + cocaine)		
Cigarettes: CBG	4.49 (2.63, 6.36)	6.70E− 05
**Full additive model**		
svyglm(PC1 raw anomalies ~ cigarettes + AUD + PC1− Cannabinoids + analgesics + cocaine + income + 6_races)		
AIAN	10.03 (5.3, 14.75)	0.0003
PC1-Cannabinoid	0.65 (0.2, 1.11)	0.0097
Cigarettes	− 14.12 (− 18.82, − 9.42)	3.82E−06
**Full interactive model**		
svyglm(PC1 raw anomalies ~ cigarettes * AUD * PC1-cannabinoids + analgesics + cocaine + income + 6_races)		
Cocaine	106.67 (24.71, 188.64)	0.0186
PC1-Cannabinoid	3.01 (0.47, 5.54)	0.0303
Cigarettes: AUD	320.87 (26.47, 615.27)	0.0446
AUD: PC1-Cannabinoid	− 34.59 (− 66.35, − 2.83)	0.0447
AUD	− 82.77 (− 148.26, − 17.29)	0.0218
Asian	− 23.31 (− 40.72, − 5.89)	0.0159
Cigarettes	− 35.71 (− 50.08, − 21.35)	8.11E−05

Final robust generalized linear regression models for CAR Dataset.

**Table 3:** Robust IPW-weighted generalized regression analyses on PC1 for ETOPFA-adjusted congenital anomaly data.

Parameter	Estimate (C.I.)	P-Value
**ETOPFA data**		
Drugs—additive model		
svyglm(PC1_ETOPFA_anomalies ~ cigarettes + AUD + mrjmon + Analgesics + cocaine + MHY)		
Cannabis	1.56 (0.51, 2.6)	0.0071
Cigarettes	− 13.19 (− 17.62, − 8.75)	3.83E−06
Drugs—interactive model		
svyglm(PC1_ETOPFA_anomalies ~ cigarettes * AUD * mrjmon + Analgesics + cocaine + MHY)		
AUD	98.08 (58.27, 137.89)	5.81E−05
AUD: Cannabis	21.02 (2.59, 39.44)	0.0345
Cigarettes: AUD	− 229.76 (− 329.02, − 130.5)	0.0001
Cannabinoids—additive model		
svyglm(PC1_ETOPFA_anomalies ~ cigarettes + AUD + THCRt + CBGRt + CBCRt + analgesics + cocaine)		
CBG	2.94 (1.39, 4.48)	0.0010
CBC	− 1.53 (− 2.67, − 0.39)	0.0146
Cigarettes	− 13 (− 17.52, − 8.48)	7.33E−06
Cannabinoids—interactive model		
svyglm(PC1_ETOPFA_anomalies ~ cigarettes * aUD * THCRt * CBGRt * CBCRt + analgesics + cocaine)		
Cigarettes: CBG	4.53 (2.67, 6.39)	6.70E−05
CBG	3.49 (0.44, 6.53)	0.0338
Cigarettes: THC: CBC	3.78 (0.23, 7.34)	0.0473
Full additive model		
svyglm(PC1_ETOPFA_anomalies ~ cigarettes + AUD + PC1_Cannabinoids + analgesics + cocaine + income + 6_races)		
PC1_Cannabinoid	0.64 (0.22, 1.06)	0.0063
Cigarettes	− 12.33 (− 16.59, − 8.07)	5.73E−06
Full interactive model		
svyglm(PC1_ETOPFA_anomalies ~ cigarettes * AUD * PC1_cannabinoids + analgesics + cocaine + income + 6_races)		
PC1_Cannabinoid	0.64 (0.22, 1.06)	0.0063
Cigarettes	− 12.33 (− 16.59, − 8.07)	5.73E−06
Cannabinoids full additive model		
svyglm(PC1_ETOPFA_anomalies ~ cigarettes + THC + CBG + CBC + AUD + Analgesics + cocaine + income + 6_races)		
CBG	3.94 (2.62, 5.25)	6.46E−06
AIAN	19.58 (12.65, 26.52)	1.46E−05
Cocaine	94.7 (12.92, 176.47)	0.03337
AUD	− 26.81 (− 45.85, − 7.78)	0.01141
CBC	− 2.8 (− 4.48, − 1.11)	0.00366
Cigarettes	− 11.53 (− 15.82, − 7.24)	2.79E−05
Cannabinoids full interactive model		
svyglm(PC1_ETOPFA_anomalies ~ cigarettes * THC * CBG * CBC + AUD + Analgesics + cocaine + income + 6_races)		
AIAN	11.36 (8.14, 14.58)	6.01E−07
CBG	3.8 (1.74, 5.86)	0.0015
Cocaine	78.22 (7.94, 148.49)	0.0401
Cigarettes: CBC	− 14.08 (− 24.26, − 3.9)	0.0128
Cigarettes	− 73.02 (− 115.67, − 30.38)	0.0029
Asian	− 19.81 (− 29.84, − 9.77)	0.0008

Final robust generalized linear regression models for ETOPFACAR Dataset.

**Table 4:** Robust IPW-weighted generalized regression analyses on PC1 for selected cancer incidence data.

Parameter	Estimate (C.I.)	P-value
**Drugs—additive model**		
svyglm(PC1-cancer ~ cigarettes + AUD + Cannabis + analgesics + cocaine + MHY)		
Cannabis	1.6 (1.08, 2.12)	2.67E−07
AUD	− 22.88 (− 32.34, − 13.42)	2.02E−05
**Drugs—interactive model**		
svyglm(PC1-Cancer ~ cigarettes * AUD * cannabis + analgesics + cocaine + MHY)		
Cigarettes	54.66 (26.52, 82.8)	0.0004
Cigarettes: Cannabis	15.56 (6.58, 24.55)	0.0015
AUD	48.5 (8.04, 88.96)	0.0233
Cigarettes: AUD: Cannabis	− 140.39 (− 258.36, − 22.41)	0.0243
Cigarettes: AUD	− 655.34 (− 1016.26, − 294.42)	0.0009
**Cannabinoids—additive model**		
svyglm(PC1-cancer ~ cigarettes + AUD + THC + CBG + CBC + Analgesics + cocaine)		
THC	2.4717 (1.81, 3.13)	2.23E−09
CBC	− 1.7349 (− 2.78, − 0.69)	0.0020
**Cannabinoids—interactive model**		
svyglm(PC1-cancer ~ cigarettes * AUD * THC * CBG * CBC + Analgesics + cocaine)		
Cigarettes: THC: CBG: CBC	1.47 (0.9, 2.04)	7.84E−06
Cigarettes: CBG: CBC	9.18 (5.35, 13.01)	2.70E−05
Cigarettes: CBG	36.19 (20.55, 51.83)	4.56E−05
THC: CBG: CBC	− 0.17 (− 0.31, − 0.04)	0.0133
CBG	− 7.44 (− 11.98, − 2.9)	0.0025
CBG: CBC	− 1.74 (− 2.71, − 0.76)	0.0012
**Full additive model**		
svyglm(PC1-cancer ~ cigarettes + AUD + PC1-Cannabinoids + analgesics + cocaine + income + 6_races)		
Asian	10.72 (8, 13.44)	9.88E−10
African	0.46 (0.32, 0.59)	2.64E−08
PC1-Cannabinoid	0.37 (0.25, 0.48)	1.32E−07
Caucasian	5.38 (3.31, 7.45)	6.89E−06
Hispanic	0.36 (0.17, 0.55)	0.0005
**Full Interactive Model**		
svyglm(PC1-cancer ~ cigarettes * AUD * PC1-cannabinoids + analgesics + cocaine + income + 6_races)		
Asian	10.24 (7.77, 12.71)	3.15E−10
African	0.48 (0.35, 0.6)	2.73E−09
Caucasian	4.86 (3.06, 6.66)	3.99E−06
Cigarettes: PC1-Cannabinoid	5.38 (3.01, 7.74)	5.85E−05
Hispanic	0.29 (0.11, 0.46)	0.0024
PC1-Cannabinoid	− 0.79 (− 1.31, − 0.27)	0.0050
**Cannabinoids full additive model**		
svyglm(PC1-cancer ~ cigarettes + THC + CBG + CBC + AUD + Analgesics + cocaine + income + 6_races)		
THC	0.97 (0.75, 1.19)	5.15E−11
Asian	9.2 (6.75, 11.65)	3.46E−09
African	0.41 (0.28, 0.53)	5.71E−08
Caucasian	4.32 (2.43, 6.22)	5.35E−05
Hispanic	0.28 (0.09, 0.46)	0.0051
**Cannabinoids full interactive model**		
svyglm(PC1-cancer ~ cigarettes * THC * CBG * CBC + AUD + Analgesics + cocaine + income + 6_races)		
Asian	7.31 (5.31, 9.31)	1.52E−08
African	0.37 (0.26, 0.48)	8.87E−08
THC: CBG: CBC	4.93 (2.83, 7.03)	4.67E−05
THC	70.96 (40.45, 101.47)	5.22E−05
Caucasian	2.79 (1.51, 4.06)	0.0001
THC: CBG	21.27 (11.19, 31.35)	0.0002
THC: CBC	16.76 (7.95, 25.57)	0.0006
Cigarettes: THC: CBC	− 89.35 (− 126.43, − 52.27)	3.14E−05
Cigarettes: THC: CBG	− 115.05 (− 161.96, − 68.14)	2.42E−05
Cigarettes: THC: CBG: CBC	− 26.26 (− 36.06, − 16.46)	6.06E−06
Cigarettes: THC	− 380.98 (− 519.06, − 242.9)	3.69E−06

Final robust generalized linear regression models for cancer Dataset.

Table 3 is set out like Table 2 but also includes additive and interactive comprehensive models for cannabinoids. Terms including cannabinoids are positive and very highly significant (from β-est. = 3.94 (2.62, 5.25), P = 6.46 × 10^–6^ in model seven) in all eight models and in each case the overall effect of rising cannabinoid exposure is positive overall.

Table 4 is structured similarly to Table 3. In each of the eight final robust models illustrated terms including cannabinoids are positive and highly significant (from β-est. = 0.97 (0.75, 1.19), P = 5.15 × 10^–11^ in model seven) and the overall effect of cannabinoids is positive in seven of the eight models (the exception being model eight).

Hence in these robust regression models many terms involving cannabinoids are again noted to be positive and highly significant and the effects of cannabinoids are strongly positive across this model series overall.

Supplementary Table 10 and Table 5 list the E-Values which may be drawn from the linear and instrumental variable regression model respectively. Minimum E-Values from linear models are noted to range from 1.21 to 11.25, with median, mode and interquartile ranges of 3.05, 3.44 (1.58, 5.86, Supplementary Table 10). Similar statistics for the minimum E-Values from the instrumental variable regression models are 10.96, 12.01 (2.23, 1250, Table 5) so that the minimum E-Values arising from the more sophisticated type of regression are significantly larger than those originating from simple linear regression (Wilcoxson’s W = 179.5, P = 0.0011).

**Table 5:** E-values of instrumental variable regression models.

Parameter	Estimate (C.I.)	E-Value
**RAW RATES**		
Drugs—additive model		
Cannabis	1.17 (0.3, 2.04)	5.02, 1.93
Drugs—interactive model		
Cigarettes: Cannabis	19.28 (13.34, 25.21)	2.09E + 08, 7.18E + 04
AUD: Cannabis	69.54 (42.68, 96.4)	1.71E + 29, 1.20E + 18
Cannabinoids—additive model		
CBG	0.95 (0.3, 1.6)	4.04, 1.94
Cannabinoids—interactive model		
Cigarettes: CBG	3.67 (2.77, 4.56)	48.91, 22.14
Full additive model		
PC1-Cannabinoids	0.6 (0.33, 0.87)	3.03, 2.12
Full interactive model		
PC1-Cannabinoids	4.37 (2.97, 5.77)	213.43, 47.55
**ETOPFA DATA**		
Drugs—additive model		
Cannabis	1.29 (0.4, 2.17)	5.51, 2.17
Drugs—interactive model		
Cigarettes: AUD: Cannabis	73.95 (48.33, 99.57)	2.13E + 26, 2.14E + 17
Cannabinoids—additive model		
CBG	3.14 (1.74, 4.54)	37.77, 9.79
Cannabinoids—interactive model		
Cigarettes: CBC	2.31 (1.5, 3.11)	17.25, 7.75
CBG	7.38 (4.18, 10.57)	2.16E + 03, 105.18
CBG: CBC	0.98 (0.49, 1.47)	4.51, 2.57
Full additive model		
PC1-Cannabinoids	0.68 (0.39, 0.97)	3.15, 2.23
Full interactive model		
PC1-Cannabinoids	5.4 (3.79, 7)	595.814, 109.21
Cannabinoid Full Additive Model		
CBG	4.21 (2.91, 5.5)	176.69, 44.16
**CANCER DATA**		
Drugs—additive model		
Cannabis	0.45 (0.2, 0.69)	2.83, 1.87
Drugs—interactive model		
Cigarettes: Cannabis	14.84 (10.88, 18.79)	2.82E + 07, 3.53E + 05
Cannabinoids—additive model		
THC	1.98 (1.61, 2.36)	18.58, 12.01
Cannabinoids—interactive model		
CBC	12.78 (6.96, 18.59)	1.53E + 07, 1.14E + 04
THC: CBC	3.02 (1.33, 4.71)	84.47, 9.91
THC: CBG: CBC	0.6 (0.17, 1.04)	3.63, 1.76
CBG	7.27 (0.46, 14.09)	1.66E + 04, 2.99
CBG: CBC	1.79 (0.1, 3.49)	18.01, 1.53
Full additive model		
PC1-Cannabinoid	0.35 (0.29, 0.41)	2.51, 2.25
Full interactive model		
Cigarettes: PC1-Cannabinoid	6.63 (4.8, 8.46)	1.45E + 04, 1.25E + 03
Full additive model with cannabinoids		
THC	1.48 (1.19, 1.77)	14.18, 9.43
Full interactive model with cannabinoids		
CBG	10.6 (4.39, 16.81)	7.52E + 06, 1.08E + 03
THC: CBG: CBC	3.81 (1.54, 6.08)	4159.71, 17.54
CBG: CBC	2.47 (0.93, 4)	67.03, 7.00
THC: CBG	16.09 (5.82, 26.36)	1.89E + 10, 9.37E + 03
CBC	7.52 (2.44, 12.59)	9.16E + 05, 65.62
THC	46.13 (11.12, 81.15)	8.05E + 28, 1.73E + 07
THC: CBC	11.15 (0.87, 21.42)	1.63E + 07, 6.57

E-Values from instrumental variable regression models.

56 minimal E-Values are listed in descending order in Supplementary Table 11 where 55 are noted to be greater than 1.25, 29/56 (51.87%) exceed five and 21 are larger than 9.0. The mean, median and modal E-Values for this series are 2.52 × 10^16^, 5.365 and 7.75 and the interquartile range is 2.17 to 44.16. The significance of these values is that 1.25 is the generally accepted cut-off for causal effects^71^ and 9.0 is the applicable E-Value for the tobacco-lung cancer relationship and is generally considered to be large^72^. Five is also considered to be a sizeable E-Value. Given that this is a comprehensive list of positive E-Values to emerge from these models this is an impressive list of minimum E-Values.

**Figure 9:**
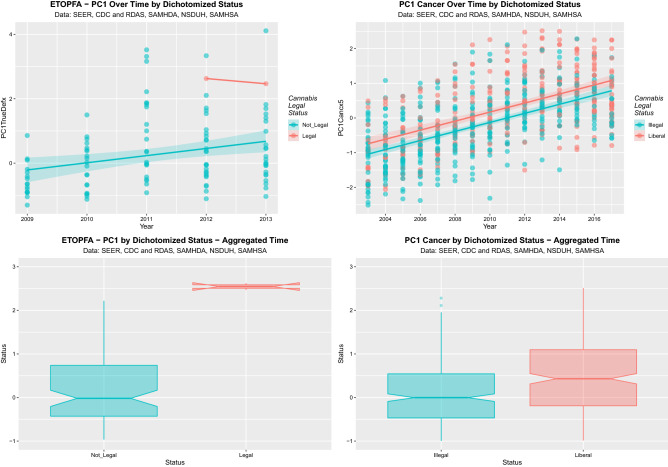
Effect of dichotomized cannabis legal status on PC1 for ETOPFA-corrected and cancer data. (**A**) Scatterplot over time for PC1 for ETOPFA-corrected anomalies for legal status dichotomized as legal cannabis states v not legal cannabis states. (**B**) Scatterplot over time for PC1 for cancer incidence dichotomized by legal status as illegal states v. liberal states. (**C**) Boxplot for PC1 for ETOPFA-adjusted congenital anomalies time-aggregated data by legal status dichotomized as in (**A**). (**D**) Boxplot for PC1 for selected cancer incidence of dichotomized legal status over aggregated time dichotomized as in (**B**). (Created in R-Studio version 1.3.1093 using ggplot2 version 3.3.2).

Supplementary Figs. 16–18 show the time dependent and box plot aggregated charts across the three domains by cannabis legal status. Strong effects are shown on dichotomization as indicated. Some of these are summarized in Fig. 9.

Final models from the time-dependent analysis of these data as continuous variables are shown in Table 6. Many highly significant effects shown including the effect of legal cannabis on the CAR data (β-est. = 2.57 (1.03, 4.11), P = 0.0014) and the effect of liberal as opposed to illegal cannabis regimes on cancer rates (β-est. = 0.58 (0.44, 0.72), P = 1.14 × 10^–15^).

**Table 6:** Effects of legal status.

Parameters			Model parameters			
Parameter	Estimate (C.I.)	P-value	R-Squared	Wald ChiSqu	dF	P
**Congenital anomaly raw data**						
lm(PC1DefectRaw ~ status)						
Medical	0.11 (− 0.41, 0.64)	0.6720	0.0968	4.93	3107	0.0030
Decriminalized	0.62 (0.08, 1.16)	0.0263				
Legal	2.57 (1.03, 4.11)	0.0014				
lm(PC1DefectRaw ~ dichotomized status)						
Liberal (v Illegal)	0.46 (0.03, 0.89)	0.0388	0.0298	4.375	1109	0.0388
lm(PC1DefectRaw ~ dichotomized status)						
Legal (v Not Legal)	2.43 (0.88, 3.98)	0.0027	0.0711	9.425	1109	0.0027
**Congenital anomaly ETOPFA-adjusted data**						
lm(PC1_ETOPFA_adjusted ~ status)						
Medical	0.16 (− 0.37, 0.68)	0.5643	0.0822	4.283	3107	0.0067
Decriminalized	0.58 (0.04, 1.11)	0.0385				
Legal	2.42 (0.87, 3.96)	0.0027				
lm(PC1_ETOPFA_adjusted ~ dichotomized status)						
Liberal (v Illegal)	0.45 (0.03, 0.88)	0.0403	0.0292	4.305	1109	0.0404
lm(PC1_ETOPFA_adjusted ~ dichotomized status)						
Legal (v not legal)	2.27 (0.72, 3.82)	0.0048	0.0620	8.272	1109	0.0048
**Cancer data**						
lm(PC1_cancer ~ status)						
Medical	0.62 (0.44, 0.8)	1.84E−11	0.0801	22.73	3746	4.31E−14
Decriminalized	0.52 (0.33, 0.7)	3.61E−08				
Legal	0.69 (0.32, 1.05)	0.00026				
lm(PC1_cancer ~ dichotomized status)						
Liberal (v Illegal)	0.58 (0.44, 0.72)	1.14E−15	0.081	67.05	1748	1.14E−15
lm(PC1_cancer ~ dichotomized status)						
Legal (v Not Legal)	0.45 (0.08, 0.83)	0.0182	0.0061	5.6	1748	0.0182

Final linear regression models for the effects of cannabis legal status in CAR, ETOPFACAR and cancer datasets.

As suggested by the dichotomous boxplots in these four figures these data can also be analyzed as categorical variables by dichotomous legal status. The CAR PC1 data may be dichotomized as illegal (-0.064 ± 0.114, mean ± S.E.M.) compared to liberal (0.395 ± 0.206; t = 1.945, df = 70.588, P = 0.056) or not legal (0.078 ± 0.106) compared to legal (2.509 ± 0.002; t = 22.76, df = 108.11, P = 9.67 × 10^–43^). The ETOPFACAR PC1 data may be dichotomized as not legal (0.273 ± 0.106) compared to legal (2.546 ± 0.081; t = 17.01, df = 7.330, P = 3.71 × 10^–7^). The cancer PC1 data may be dichotomized as illegal (− 0.277 ± 0.0.54) compared to liberal (0.189 ± 0.615; t = 5.703, df = 476.848, P = 2.06 × 10^–8^).

These results confirm formally at both continuous and categorical analysis the strong visual impression from inspection of Supplementary Figs. 16–18 and Fig. 9 that liberal cannabis regimes greatly exacerbate the rates of CAR’s, ETOPFACAR’s and cancers studied at high levels of statistical significance.

Supplementary Table 12 shows Cohen’s D as an effect size measure, the qualitative characterizations of Cohen’s D, and the applicable E-Values and P-Values for legal status metrics. The strong effects noted in the preceding graphs are confirmed here on quantitative analysis as both effect sizes and minimum E-Values.

## Discussion

The present study examined the bivariate and multivariate relationships of the principal components of five chromosomal pathologies (trisomies 13, 18, 21, the monosomy Turner syndrome and Deletion 22q11.2) and five cancers (thyroid, liver, breast and pancreatic cancer and acute myeloid leukaemia) to substance exposure, income and ethnicity covariates in a quantitative causal analysis framework. The main results of the present study were that all ten pathologies examined are rising both across time and in relation to the five cannabinoids examined [cannabis, THC, cannabinol (CBN), cannabichromene (CBC) and cannabigerol (CBG)] in a manner which was robust to adjustment in multivariable inverse probability weighted instrumental variable and robust generalized regression models. Cannabis and all four cannabinoids were significantly related to the PC1’s for chromosomal anomaly rates both before and after adjustment for ETOPFA’s and to the cancer PC1 in final regression models after full adjustment for sociodemographic factors. Large effect sizes were demonstrated for both congenital anomaly PC’s between the highest quintile of cannabis use and the third and fourth quintiles and a medium effect size was shown for the cancer PC. Large effect sizes were also demonstrated between states with legal and not legal cannabis legal regimes for chromosomal anomaly PC’s and a medium effect size was demonstrated between illegal and liberal legal paradigms for the cancer PC1. These effect sizes were accompanied by appropriately large minimal E-values of greater than five and 2.4 respectively, and small P-values (P = 9.67 × 10^–43^ and P = 2.66 × 10^–15^ respectively).

Previous studies have reported individual conditions in the young known to be associated with cannabis-related chromosomal damage: current data highlight cannabis damage on multiple human chromosomes. Live-born congenital anomaly rates are known to underestimate true rates due to both spontaneous and induced abortion of damaged foetuses. The existence of an experimentally well described threshold dose in the micromolar range^73–81^ explains both why inherited morbidity is not observed more commonly at present after prenatal exposure and why rates jump abruptly with the increased use, availability and concentration implicit under the legalization paradigm as shown clearly by epidemiological data from Colorado, Canada, Hawaii and Australia^8,10,37,82,83^.

It is intriguing to note the variety of chromosomal-toxic mechanisms which are implied by the present results. Whereas the trisomy/monosomy (Turner) syndromes are presumably related to chromosomal mis-segregation errors^4^, horizontal transversions and gene amplifications on chromosome 12 in testicular cancer^43–46,84,85^ and deletion of the short arm of chromosome 22 in Deletion 22q11.2 signify multiple pathways to major chromosomal pathology.

This report studied five congenital chromosomal anomalies and five cancers. Dose–response effects were noted for many associations which were independently significant for cannabis, THC, cannabichromene and cannabigerol and these relationships were preserved after adjustment for estimated ETOPFA rates which is a preponderant effect especially for chromosomal anomalies which are terminated prior to birth at high rates. All ten disorders were noted to rise strongly over time and in relationship to cannabis and the cannabinoids THC, cannabigerol, cannabinol and cannabichromene. Strong effects by quintile of cannabis exposure were noted which were also reflected in the impact of cannabis legal status. Many effect sizes were noted to be strong with many Cohen’s D’s above 1.4, where relationships above 0.8 being typically described as being strong^86^. The pooled disorder-cannabinoid relationship satisfied formal criteria of causality as assessed by inverse probability weighting of robust marginal structural models with P-values significant from 2.8 × 10^–7^ and 55/56 e-Values being greater than 1.25 which is the causal threshold and 30/56 minimum e-Values being greater than 5 which is relatively large. The individual disorders are also the subject of separate space–time and causal inference analyses which are presently being prepared.

The findings are prominent for showing a dramatic rise from the fourth to the fifth quintile of cannabis use and a reflection of a similar kind when considering the cannabis legal paradigm. This is reminiscent of a similar finding for 34 defects recently published which also showed a major jump from the fourth to fifth quintile^39^. This is concerning because it directly reflects the well described exponential dose response which has been found in many cannabinoid genotoxicity studies and in many studies of the pharmacology of cannabinoids generally^14,26,59–61^.

A direct corollary of this bench to bedside parallelism is that as the community moves steadily into higher echelons of cannabis use the genotoxic sequelae will be unprecedentedly magnified—in coming generations. It is this multi-generational and transgenerational aspect of cannabinoid genotoxicity which is of particular concern in the context of disproportionate dose-exposure escalation.

Cannabis use amongst young adults has been unanimously linked in four of four studies with the subsequent development of testicular cancer^43–46^. Testicular cancer is interesting in that 90% of cases involve the formation of an isochromosome 12, and in the remainder an internal intra-chromosomal amplification of parts of the long arm of chromosome 12 occurs so that the relative gene dosage is increased under both scenarios^40^. Cannabis exposure—testicular oncogenesis dose–response effects have been described in several epidemiological series^43–45^. Testicular cancer is believed to arise from pro-oncogenic germ stem cell mutations which occur during in utero life which are subsequently activated by the hormonal surge of pubertal development^40–42^. In the case of this tumour therefore cannabis accelerates the subclinical pro-oncogenic phase from several decades to just a few years.

Most particularly, the present demonstration of cannabinoid-linked genotoxicity applying to over 500 MB of the human genome accommodated on chromosomes 12, 13, 18, 21 and X clearly indicates that mechanisms exist in man linking in vitro genotoxic effects to clinical effects. Hence it becomes plausible to link the 21 congenital defects noted in Hawaii with cannabis-only exposure^37^, the 13 congenital anomalies noted in Australia^38^, limb defects noted in France and Germany^87,88^, 29% increase in total congenital anomalies listed in Colorado^8^ a tripling of total birth defects in the high-cannabis using areas of Canada^10^ and 34 congenital anomalies in USA^39^ to transgenerational cannabinoid-induced genotoxic mechanisms. Evidence presented herein also implicates the PC1 of five cancers seen clinically including acute myeloid leukaemia which has been previously documented^89,90^ and breast cancer which is the most common cancer of all across USA with 279,100 cases expected in 2020^91^. Cannabis has previously been linked with both the induction and promotion of liver fibrosis and cirrhosis^92,93^ and with hepatic neocarcinogenesis by numerous mechanisms^94^. It has not been previously linked with the other cancers studied to our knowledge.

Central to any discussion of genotoxic mechanisms of cannabis are considerations of the biological mechanisms by which it mediates chromosomal derangements and disruptions. Cannabis and the cannabinoids THC, cannabinol, cannabidiol and cannabinol have been shown to be toxic to oocytes^6^, sperm^24,95,96^, chromosomes^96^, the bases of DNA^97^ and epigenetic regulation both by DNA methylation^4,19,20,22–24,98^ and histone formation^16,99^. Cannabidiol and cannabidivarin in low doses have been shown to directly oxidize DNA bases which is a highly oncogenic and mutagenic action^97^. Cannabinoids have long been recognized to reduce the synthesis of major macromolecules of life including DNA, RNA proteins and histones^13,18,60,99–104^. Reduction in the linker histone H1 has recently been shown to comprise a major oncogenic mechanism by making genes more accessible for transcription^105^. One of the proteins whose synthesis is impeded is tubulin^4,16^. Tubulin polymerization has many key roles in side the cell including the formation of the microtubules of the mitotic spindle and the molecular skeleton of axons, cilia, centrosomes and flagella^106^. Deranged microtubular function has been linked with chromosomes sliding off the mitotic spindle in anaphase and the formation of micronuclei^4,6,7,107^ which are described as being a major generator of the genetic chaos of cancer^4,107–118^. Indeed, just as histones undergo post-translational modifications tubulin has also been shown to undergo post-translational modifications which target the tubulin monomers for different subcellular destinations^105^. Errors in this “tubulin code” have been linked to disorders of flagellar function so that sperm are not able to swim normally in a linear trajectory and go round in circles and fail to correctly target oocytes which is believed to be a potentially significant cause of male infertility^105^. Hence whilst for descriptive purposes it is useful to describe cannabinoid-related molecular aberrations in various stratified layers and subcellular compartments, it seems likely that in reality the various layers are intimately crosslinked and molecularly interdependent^53,54,119–121^. Unfortunately space precludes a more detailed discussion in this forum but many of the important issues have been addressed elsewhere^4,8–12,14,15,19,20,23,24,38,39,61,102,122,123^.

We feel that our results are widely generalizable for several reasons. The cannabinoid-genotoxicity relationship fulfils most of the Hill criteria for causality including of strength of association, consistency among studies, specificity, temporality, coherence with known data, biological plausibility, dose–response relationship, analogy with situations internationally and a rich experimental research base^124^. Naturally further details will be provided in manuscripts addressing each of these issues individually including consideration in the native space–time data context and formal causal inferential analyses. As noted many of the above findings have been replicated several times elsewhere particularly with relation to the congenital chromosomal anomalies^8,10,37,38^. These results are based on the best data available globally. Since causal relationships were demonstrated herein we would expect these relationships to be maintained wherever adequate data quality allows their assessment.

The techniques of causal inference are well satisfied by these results. For comparison, one notes that the E-Value, or Expected value for the tobacco-lung cancer association is 9.0. As noted 21/56 (37.5%) of the E-Values reported herein are above this cut-off and 29/56 (51.87%) exceed 5 which is also a sizeable E-Value.

The E-Value is the value of association required of some extraneous hypothetical unmeasured confounder covariate with both the exposure of concern and the outcome of interest to explain away the observed results. With such high results as are reported in our present paper such a confounder seems most unlikely. Inverse probability weighting is the technique of choice to correctly weight an observational study and turn it into a pseudo-randomized study from which causal inferences can appropriately be drawn. The classic concern with observational studies is that one is in actually comparing “apples with oranges”. The use of inverse probability weighting ensures that everything is “apples” as it were. It is therefore important to appreciate that, although it is true that our study uses several multiple regression techniques, the extensive use of the techniques of quantitative causal inference particularly inverse probability weighting and E-values are the appropriate tools with which to address causal relationships and formally draw causal inferences.

Our study has many strengths including the use of advanced statistical techniques and the formal techniques of causal inference. Its limitations include that we have not had space here to address each disorder separately as those analyses are destined for other manuscripts. In common with all epidemiological studies we do not have individual patient-level data available to us. It is also relevant to observe that the quantitative criteria fulfilled by the analytical procedures in this study are those of causal inference in epidemiology and are widely acknowledged in the discipline. However this is not the same thing as the formal assessment of causality as is done in the controlled experimental laboratory setting, however given that it is not expected to ever be ethical to conduct clinical trials of prenatal exposures these various analytical procedures are the next best thing which can be achieved in clinical populations. Having said that we heartily endorse ongoing research into the many mechanisms of cannabinoid genotoxicity for the same reason that the mechanisms of action of thalidomide continue to be investigated experimentally to better understand its pathophysiology, to remediate its damage, and to develop new lead compounds for novel clinical applications in cancer medicine and elsewhere. As patients can be confused about the impact of early gestational cannabis exposure on developing pregnancies we advocate for the development of a reliable biomarker to quantitate exposure and denominate future studies^11^.

Some of the statistical techniques used herein also have theoretical limitations. For example it has been noted that inverse probability weighting cannot be used if all the subjects of a certain class at any time during the study must receive a certain exposure condition^125^. Inverse probability weighting has also been observed not to work well with small samples^126^. And its use for dealing with missing data also has methodological weaknesses^127^. However these conditions were not observed in the present dataset and inverse probability was not used to address missing data in these analyses. The interpretation of E-Values is necessarily always subjective and relies on some background knowledge of the subject. For example if an E-Value is reported as five then the judgement must be made as to whether confounding variables are likely to exist which correlate with both the exposure of interest and the outcome of concern of the calculated magnitude to explain away an apparently causal effect. In the present study with median and modal minimum E-Values of 5.65 and 7.75 this seems quite unlikely. Whilst the use of principal components is a common analytical device it can never substitute for detailed investigations of each identified syndrome separately and in detail. For this reason detailed causal modelling and spatiotemporal analyses are indicated on each of the pathologies identified to further investigate the effects reported herein in aggregate.

This report is intended as an introductory overview only and serves the purpose of introducing the subject to readers’ consideration and detailed geospatial and causal inference studies of many congenital anomalies and cancers are indicated to further explore these findings, issues which are indeed the subject of other recent papers^4,8,10,52,82,83,128–133^ and current manuscripts. In conclusion this study of recent US data not only confirms previous findings linking cannabis use with congenital and chromosomal anomalies, but it shows that those impacts are significant at the public health level, likely account for much of their recent rise, explain the worrisome discontinuity and jump from the fourth to the fifth quintiles for cannabis exposure and are consistent both with a rich experimental database and experience from other countries^8,10,37,38^. Since findings implicate over 500 MB of the human genome this directly explains the association of cannabis use with many other congenital anomalies and heritable carcinogenesis previously reported. In the context of an exponential dose–response curve for metabolically-genotoxically- and epigenetically-mediated cannabinoid-induced genotoxicity^14,26,59–61^ the rising level of cannabis use induced by cannabis legalization and its severe sequaelae would appear to be more than sufficient contraindication to continued relaxation of the laws surrounding cannabis, risks further compounded by increasingly described heritable neurotoxicity^128,132,134–139^.

## Methods

### Data

Data on US birth defect rates was downloaded from the National Birth Defect Prevention Network (NBDPN) from CDC Atlanta Georgia website annual reports^63^. Estimated early termination of pregnancy for anomaly (ETOPFA) rates by birth defect type were taken as an average of a composite score from several Australian and USA published series shown as Supplementary Table 13^64–66^. The rate of change over time of these ETOPFA rates was taken from the only longitudinal annual series of ETOPFA rates which could be identified which was the Western Australian series for Down syndrome (Supplementary Table 14^140^).

Age-adjusted state cancer data was taken from the National Program of Cancer Registries (NPCR) and Surveillance Epidemiology and End Results (SEER) Incidence dataset US Cancer Statistics Public Use Database 2019 submission (2001–2017)^62^.

Drug use data by state was taken from the Restricted Data Analysis System (RDAS) from the annual National Survey of Drug Use and Health (NSDUH) of the Substance Abuse and Mental Health Data Archive (SAMHDA) from Substance Abuse and Mental Health Services Administration (SAMHSA)^67^. Intensity of cannabis use data by ethnicity was taken from the RDAS, NSDUH at SAMHDA. Concentration of various cannabinoids nationally was taken from published reports from the Drug Enforcement Agency (DEA)^68–70^. Median household income data was downloaded from the US Census Bureau using tidycensus package in R^141^. Cannabis legal status in each state was taken from an Internet search^142^.

### Derived data

Intensity of ethnic-specific cannabis use was multiplied by state monthly cannabis use and the THC concentration in Federal seizures to derive an estimate of ethnic THC exposure at state level. Quintiles of cannabis use were derived by dividing the states for each year into five groups for cannabis use with details as shown in Supplementary Table 15. State-based cannabinoid exposure was calculated by multiplying the state levels of monthly cannabis use by the applicable cannabinoid concentration in Federal seizures. Chromosomal anomalies are extensively screened for prenatally and subject to high rates of early termination of pregnancy for anomaly (ETOPFA). Accordingly ETOPFA-corrected congenital anomaly rates were calculated by dividing the observed anomaly rate in any year by the composite ETOPFA rate for that anomaly multiplied by the fraction of ETOPFA for that year obtained from the Western Australian longitudinal series.

### Statistics

Data was processed using R version 4.0.2 and R-Studio 1.3.1093 in October 2020. Data are listed as mean ± standard error of the mean (S.E.M.). Data was manipulated using dplyr and graphs were drawn using ggplot2, both from the tidyverse suite^143^. Correlograms were drawn using the packages corrplot and corrgram from R^144,145^. Linear regression was performed using R-Base. Two-step instrumental variable regression was performed using package AER^146^. Robust inverse probability weighted regression was performed using the survey package^147^ with State as the identifying variable. In all cases initial models were serially reduced manually by the deletion of the least significant term by the classical technique. The overall direction of models with rising cannabinoid exposure was determined by matrix multiplication with other covariates held constant at their means. Effect size was quantitated using Cohen’s D from the effsize package^86^. Principal Components (PC’s) were calculated using the psych package and the number of PC’s required was determined formally using a Scree plot and factor analysis^148^. Inverse probability weights were calculated using the ipw package^149^. E-Values were calculated using the E-Value package^149^. T-tests were two-tailed. P < 0.05 was considered significant throughout.

### Ethics

The Human Research Ethics Committee of the University of Western Australia approved this study on 7th January 2020 RA/4/20/7724.

## Supplementary Information

Supplementary Information.

## Data Availability

Raw data including the software computing script accompanying this article have been made available online in the Mendeley data repository and may be found at 10.17632/xwrkp6kjd9.1.
