# Supplementary material for: Epidemiological overview of multidimensional chromosomal and genome toxicity of cannabis exposure in congenital anomalies and cancer development
Source: Sci Rep. 2021 Jul 6;11:13892. doi: 10.1038/s41598-021-93411-5 (PMC8260794; doi:10.1038/s41598-021-93411-5)
Supplement: Supplementary file 1 — Supplementary Information. [file 41598_2021_93411_MOESM1_ESM.pdf]

Epidemiological Overview of Multidimensional Chromosomal and Genome Toxicity of Cannabis Exposure in Congenital Anomalies and Cancer Development

Supplementary Material

Albert Stuart Reece & Gary Kenneth Hulse

Supplementary Tables

Supplementary Table List

Table	Subject
-	-
Supplementary Table 1	Cannabis Use by Quintiles
Supplementary Table 2	Details of PC1 for Raw Congenital Anomaly Rates
Supplementary Table 3	Details of PC1 for ETOPFA-Adjusted Congenital Anomaly Rates
Supplementary Table 4	Details of PC1 for Cannabinoid Exposure
Supplementary Table 5	Details of PC1 for Selected Cancer Incidence
Supplementary Table 6	Linear Regressions on Quintiles
Supplementary Table 7	Instrumental Regression on PC1 of Raw Congenital Anomaly Data
Supplementary Table 8	Instrumental Regression on PC1 of ETOPFA-Adjuted Congenital Anomaly Data
Supplementary Table 9	Instrumental Regression on PC1 on Selected Cancer Incidence Data
Supplementary Table 10	E-Values of Linear Models
Supplementary Table 11	Minimum E-Values List
Supplementary Table 12	Effect Sizes, E- and P-Values for Legal Status Analyses
Supplementary Table 13	ETOPFA Rates
Supplementary Table 14	Historical Sequence of ETOPFA Rates for Downs Syndrome in Western Australia
Supplementary Table 15	State Composition of Cannabis Use Quintiles

Supplementary Table 1 – Cannabis Use by Quintiles

**Cannabis Use Quintile**	**Estimate (95% C.I.)**
Quintile 1	0.060 (0.057, 0.063)
Quintile 2	0.067 (0.064, 0.070)
Quintile 3	0.072 (0.069, 0.074)
Quintile 4	0.085 (0.081, 0.088)
Quintile 5	0.114 (0.108, 0.119)

Supplementary Table 1 shows the ranges of percent cannabis exposure in each of the five cannabis use quintiles as fractions of the population exposed. The 95% confidence interval is also shown.

Supplementary Table 2 – PC1 Raw Rates

Disorder	PC1
Downs Syndrome, Trisomy 21	0.84
Trisomy 18	0.80
Tisomy 13	0.76
Deletion 22q11.2	0.78
Turners Syndrome	0.89
Test PC's =1 Sufficient:	
Root Mean Square Residuals	0.11
Chi Squared	169.83
P-Value	4.05E-39

Supplementary Table 2. This table provides principal component weights and other details for PC1 for the raw congenital anomaly rates.

Supplementary Table 3 – PC1 ETOPFA-Adjutsed Rates

Disorder	PC1
Downs Syndrome, Trisomy 21	0.89
Trisomy 18	0.86
Tisomy 13	0.83
Deletion 22q11.2	0.71
Turners Syndrome	0.90
Test PC's =1 Sufficient:	
Root Mean Square Residuals	0.11
Chi Squared	170.96
P-Value	2.29E-39

Supplementary Table 3. This table provides details of the principal component PC1 weights and other metrics for the ETOPFA-adjusted congenital anomaly rates.

Supplementary Table 4 – Cannabinoid Exposure

**Disorder**	**PC1**
THC	0.98
CBC	0.93
CBN	0.95
CBG	0.99
Test PC's =1 Sufficient:	
Root Mean Square Residuals	0.11
Chi Squared	170.96
P-Value	2.29E-39

Supplementary Table 4. This table illustrates the weights and other details from the principal component PC1 study of cannabinoid exposures.

Supplementary Table 5 – PC1 Cancer Rates

**Disorder**	**PC1**
Liver	0.65
Thyroid	0.67
Pancreas	0.82
AML	0.60
Breast	0.61
Test PC's =1 Sufficient:	
Root Mean Square Residuals	0.11
Chi Squared	170.96
P-Value	2.29E-39

Supplementary Table 5. This table provides weights and other details for the principal component PC1 of the five cancers chosen.

Supplementary Table 6 – Linear Regression of Quintile Data

Parameters			Model Parameters			
Parameter	Estimate (C.I.)	P-Value	R-Squared	Wald ChiSqu.	dF	P
Cannabis Use Quintile						
lm(PC1AnomalyRaw ~ Quintile)						
Quintile 2	-0.79 (-1.31, -0.27)	0.0034	0.3760	17.57	4,106	4.34E-11
Quintile 3	-1.32 (-1.88, -0.76)	1.04E-05				
Quintile 4	-0.89 (-1.44, -0.33)	0.0022				
Quintile 5	0.98 (0.36, 1.59)	0.0024				
lm(PC1AnomalyRaw ~ Dichotomized Quintiles)						
Highest_Quintile	1.76 (1.22, 2.29)	3.81E-09	0.2671	41.1	1,109	3.81E-09
**ETOPFA-Corrected Anomaly Data**						
lm(PC1_ETOPFA_Adjusted ~ Quintile)						
Quintile 2	-0.71 (-1.25, -0.16)	0.0121	0.3041	13.02	4,106	1.17E-08
Quintile 3	-1.19 (-1.77, -0.6)	1.32E-04				
Quintile 4	-0.76 (-1.34, -0.18)	0.0120				
Quintile 5	0.9 (0.26, 1.55)	0.0072				
lm(PC1_ETOPFA_Adjusted ~ Dichotomized Quintiles)						
Highest_Quintile	1.59 (1.04, 2.14)	1.15E-07	0.2211	32.23	1,109	1.14E-07
**Cancer Data**						
lm(PC1_Cancer ~ Quintile)						
Quintile 2	0.18 (-0.04, 0.4)	0.105	0.656	14.18	4,745	3.69E-11
Quintile 3	0.44 (0.22, 0.66)	9.33E-05				
Quintile 4	0.58 (0.36, 0.8)	2.46E-07				
Quintile 5	0.73 (0.51, 0.95)	8.67E-11				
lm(PC1_Cancer ~ Dichotomized Quintiles)						
Highest_Quintile	0.43 (0.26, 0.61)	1.69E-06	0.0289	23.28	1,748	1.69E-06

Supplementary Table 6. This table presents the results of various linear regression models for quintiles of cannabis exposure.

Parameters			Model Parameters				
Parameter	Estimate (C.I.)	P-Value	S.D.	R-Squared	Wald ChiSqu.	dF	P
Drugs - Additive Model							
ivreg(PC1Raw_Anomalies ~ Cigarettes + AUD + Cannabis + Analgesics + Cocaine + MHY)							
Cannabis	1.17 (0.3, 2.04)	0.010	1.0361	0.5814	39.19	4,106	<2.2E-16
Cocaine	67.48 (16.48, 118.48)	0.011					
AUD	-15.32 (-30.18, -0.46)	0.046					
Cigarettes	-13.67 (-17.01, -10.33)	1.56E-12					
Drugs - Interactive Model							
ivreg(PC1Raw_Anomalies ~ Cigarettes * AUD * Cannabis + Analgesics + Cocaine + MHY)							
Cigarettes: Cannabis	19.28 (13.34, 25.21)	5.24E-09	0.9499	0.6481	41.53	5,105	<2.2E-16
AUD: Cannabis	69.54 (42.68, 96.4)	1.69E-06					
Cocaine	101.15 (54.43, 147.86)	4.76E-05					
Cigarettes: AUD	-571.8 (-802.62, -340.98)	4.21E-06					
Cigarettes: AUD: Cannabis	-486.01 (-675.19, -296.82)	1.99E-06					
Cannabinoids - Additive Model							
ivreg(PC1Raw_Anomalies ~ Cigarettes + AUD + THC + CBG + CBC + Analgesics + Cocaine)							
CBG	0.95 (0.3, 1.6)	0.0049	1.0335	0.5835	52.36	3,107	<2.2E-16
Cocaine	54.23 (9.84, 98.63)	0.0184					
Cigarettes	-13.3 (-16.62, -9.99)	3.06E-12					
Cannabinoids - Interactive Model							
ivreg(PC1Raw_Anomalies ~ Cigarettes * AUD * THC * CBG * CBC + Analgesics + Cocaine)							
Cigarettes: CBG	3.67 (2.77, 4.56)	3.06E-12	1.0402	0.578	76.33	2,108	<2.2E-16
Cocaine	64.04 (25.02, 103.05)	3.06E-12					
Full Additive Model							
ivreg(PC1Raw_Anomalies ~ Cigarettes + AUD + PC1_ + Cannabinoids + Analgesics + Cocaine + Income + 6_Races)							
PC1_Cannabinoid	0.6 (0.33, 0.87)	3.21E-05	0.9138	0.6743	33.54	7,103	<2.2E-16
AIAN	19.55 (9.87, 29.23)	0.0001					
Cocaine	80.51 (34.61, 126.41)	0.0009					
Analgesics	-1.15 (-2.05, -0.24)	0.0145					
AUD	-18.91 (-33.75, -4.07)	0.0141					
Asian	-12.58 (-22.12, -3.04)	0.0111					
Cigarettes	-14.56 (-18.38, -10.74)	2.74E-11					
Full Interactive Model							
ivreg(PC1Raw_Anomalies ~ Cigarettes * AUD * PC1_Cannabinoids + Analgesics + Cocaine + Income + 6_Races)							
PC1_Cannabinoid	4.37 (2.97, 5.77)	1.80E-08	0.8510	0.7176	32.05	9,101	<2.2E-16
Cocaine	83.44 (41.24, 125.64)	0.0002					
Cigarettes: AUD	515.33 (244.97, 785.69)	0.0003					
Hispanic	0.66 (0.29, 1.02)	0.0007					
Caucasian	3.01 (1.15, 4.87)	0.0020					
Asian	-15.27 (-25.5, -5.03)	0.0043					
AUD	-137.04 (-203.11, -70.96)	9.52E-05					
Cigarettes: PC1_Cannabinoid	-18.55 (-25.09, -12)	2.29E-07					
Cigarettes	-43.95 (-59.02, -28.87)	1.12E-07					

Supplementary Table 7 – ivreg, ipw, PC1, CAR Data

Supplementary Table 7. This table presents detailed results for instrumental variable regression results on CAR data by substances.

Supplementary Table 8 – ivreg, ipw, PC1 - ETOPFA

Parameters			Model Parameters				
**Parameter**	**Estimate (C.I.)**	**P-Value**	**S.D.**	**R-Squared**	**Wald ChiSqu.**	**dF**	**P**
**ETOPFA DATA**							
Drugs - Additive Model							
ivreg(PC1_ETOPFA_Data ~ Cigarettes + AUD + mrjmon + Analgesics + Cocaine + MHY)							
Cannabis	1.29 (0.4, 2.17)	0.0054	1.0563	0.529	31.89	4,106	<2.2E-16
Cocaine	59.38 (7.38, 111.37)	0.0273					
AUD	-16.37 (-31.52, -1.22)	0.0365					
Cigarettes	-12.24 (-15.65, -8.83)	2.04E-10					
Drugs - Interactive Model							
ivreg(PC1_ETOPFA_Data ~ Cigarettes * AUD * mrjmon + Analgesics + Cocaine + MHY)							
Cigarettes: AUD: Cannabis	73.95 (48.33, 99.57)	1.30E-07	1.1228	0.4679	33.24	3,107	2.91E-15
Cocaine	94.64 (54.89, 134.4)	8.93E-06					
AUD: Cannabis	-11.34 (-19.82, -2.86)	0.010					
Cannabinoids - Additive Model							
ivreg(PC1_ETOPFA_Data ~ Cigarettes + AUD + THCRt + CBGRt + CBCRt + Analgesics + Cocaine)							
CBG	3.14 (1.74, 4.54)	2.57E-05	0.9692	0.6035	34.48	5,105	<2.2E-16
Cocaine	71.58 (24.25, 118.92)	0.0038					
AUD	-15.35 (-29.11, -1.59)	0.0310					
CBC	-2.18 (-3.91, -0.44)	0.0154					
Cigarettes	-11.68 (-14.81, -8.55)	5.49E-11					
Cannabinoids - Interactive Model							
ivreg(PC1_ETOPFA_Data ~ Cigarettes * AUD * THCRt * CBGRt * CBCRt + Analgesics + Cocaine)							
Cigarettes: CBC	2.31 (1.5, 3.11)	1.68E-07	0.9610	0.6102	35.43	5,105	<2.2E-16
CBG	7.38 (4.18, 10.57)	1.59E-05					
CBG: CBC	0.98 (0.49, 1.47)	0.0001					
Cocaine	51.4 (7.28, 95.52)	0.0244					
Analgesics	-1.04 (-1.94, -0.13)	0.0265					
Full Additive Model							
ivreg(PC1_ETOPFA_Data ~ Cigarettes + AUD + PC1_Cannabinoids + Analgesics + Cocaine + Income + 6_Races)							
PC1_Cannabinoid	0.68 (0.39, 0.97)	1.12E-05	0.9885	0.5875	27.11	6,104	<2.2E-16
AIAN	14.03 (3.61, 24.45)	0.0096					
Cocaine	54.2 (5.07, 103.32)	0.0329					
Analgesics	-1.09 (-2.07, -0.11)	0.0314					
AUD	-22.8 (-38.03, -7.57)	0.0041					
Cigarettes	-10.35 (-13.73, -6.97)	2.92E-08					

Supplementary Table 8. This table presents detailed results for instrumental variable regression results on CAR data by substances.

Supplementary Table 8 – ivreg, ipw, PC1 – ETOPFA (Continued)

Full Interactive Model							
ivreg(PC1_ETOPFA_Data ~ Cigarettes * AUD * PC1_Cannabinoids + Analgesics + Cocaine + Income + 6_Races)							
PC1_Cannabinoid	5.4 (3.79, 7)	2.16E-09	0.8620	0.6863	25.07	10,100	<2.2E-16
Cocaine	90.19 (45.75, 134.62)	0.0001					
Hispanic	0.65 (0.28, 1.02)	0.0009					
Cigarettes: AUD	458.38 (183.99, 732.78)	0.0015					
Caucasian	3.03 (1.14, 4.92)	0.0022					
Asian	-19.64 (-31.18, -8.1)	0.0012					
AUD: PC1_Cannabinoid	-28.57 (-44.95, -12.18)	0.0009					
AUD	-123.49 (-190.65, -56.33)	0.0005					
Cigarettes: PC1_Cannabinoid	-13.46 (-20.35, -6.56)	0.0002					
Cigarettes	-39.87 (-55.14, -24.59)	1.51E-06					
Cannabinoid Full Additive Model							
ivreg(PC1_ETOPFA_Data ~ Cigarettes + AUD + THC + CBG + CBC + Analgesics + Cocaine + Income + 6_Races)							
CBG	4.21 (2.91, 5.5)	5.65E-09	0.8539	0.6922	31.92	8,102	<2.2E-16
AIAN	21.77 (12.28, 31.26)	1.83E-05					
Cocaine	95.8 (52.32, 139.28)	3.66E-05					
Analgesics	-1.16 (-2.01, -0.32)	0.0081					
AUD	-19.8 (-33.68, -5.93)	0.0062					
Asian	-13.06 (-21.93, -4.19)	0.0048					
CBC	-3.01 (-4.57, -1.45)	0.0003					
Cigarettes	-13.07 (-16.63, -9.5)	1.17E-10					
Cannabinoid Full Interactive Model							
ivreg(PC1_ETOPFA_Data ~ Cigarettes * THC * CBG * CBC + AUD + Analgesics + Cocaine + Income + 6_Races)							
Cocaine	110.05 (61.98, 158.11)	1.91E-05	0.9149	0.6467	23.37	9,101	<2.2E-16
Hispanic	0.59 (0.32, 0.85)	3.53E-05					
AIAN	18.25 (7.71, 28.79)	0.0010					
NHPI	120.73 (37.57, 203.88)	0.0054					
Asian	-14.04 (-24.09, -3.98)	0.0073					
AUD	-25.59 (-40.85, -10.33)	0.0014					
CBC	-5.59 (-8.26, -2.92)	8.36E-05					
CBG: CBC	-0.98 (-1.33, -0.64)	1.57E-07					
Analgesics	-2.54 (-3.41, -1.66)	1.27E-07					

Supplementary Table 8. This table presents detailed results for instrumental variable regression results on CAR data by substances.

Supplementary Table 9 – ivreg, ipwtm, PC1Cancer

Parameters			Model Parameters				
**Parameter**	**Estimate (C.I.)**	**P-Value**	**S.D.**	**R-Squared**	**Wald ChiSqu.**	**dF**	**P**
**CANCER DATA**							
Drugs - Additive Model							
ivreg(PC1_Cancer ~ Cigarettes + AUD + Cannabis + Analgesics + Cocaine + MHY)							
AUD	14.31 (9.36, 19.26)	2.46E-08	0.7494	0.3086	76.72	3,506	<2.2E-16
Cannabis	0.45 (0.2, 0.69)	0.0004					
Cigarettes	-11.15 (-12.89, -9.41)	<2.2E-16					
Drugs - Interactive Model							
ivreg(PC1_Cancer ~ Cigarettes * AUD * Cannabis + Analgesics + Cocaine + MHY)							
Cigarettes	52.51 (40.81, 64.21)	<2.2E-16	0.9201	0.4047	85.88	6,743	<2.2E-16
Cigarettes: Cannabis	14.84 (10.88, 18.79)	5.21E-13					
AUD	45.51 (23.8, 67.22)	4.43E-05					
Analgesics	-0.57 (-0.88, -0.27)	0.0002					
Cigarettes: AUD: Cannabis	-130.04 (-184.04, -76.03)	2.83E-06					
Cigarettes: AUD	-614.03 (-786.39, -441.68)	6.43E-12					
Cannabinoids - Additive Model							
ivreg(PC1_Cancer ~ Cigarettes + AUD + THCRt + CBGRt + CBCRt + Analgesics + Cocaine)							
THC	1.98 (1.61, 2.36)	<2.2E-16	0.8002	0.4332	144.1	4,745	<2.2E-16
Analgesics	-0.52 (-0.81, -0.22)	0.0006					
AUD	-8.01 (-12.36, -3.66)	0.0003					
CBC	-1.09 (-1.6, -0.58)	3.16E-05					
Cannabinoids - Interactive Model							
ivreg(PC1_Cancer ~ Cigarettes * AUD * THCRt * CBGRt * CBCRt + Analgesics + Cocaine)							
CBC	12.78 (6.96, 18.59)	1.90E-05	0.7334	0.5239	59.87	14,735	<2.2E-16
THC: CBC	3.02 (1.33, 4.71)	0.0005					
Cocaine	16.82 (6.62, 27.02)	0.0013					
THC: CBG: CBC	0.6 (0.17, 1.04)	0.0070					
CBG	7.27 (0.46, 14.09)	0.0368					
CBG: CBC	1.79 (0.1, 3.49)	0.0382					
Cigarettes: THC: CBG	-16.65 (-29.56, -3.74)	0.0117					
Cigarettes: THC	-85.3 (-137.59, -33)	0.0015					
Cigarettes: THC: CBG: CBC	-5.79 (-9.21, -2.38)	0.0009					
Analgesics	-0.56 (-0.84, -0.28)	0.0001					
AUD	-8.69 (-12.96, -4.41)	7.53E-05					
Cigarettes: THC: CBC	-32.75 (-48.79, -16.71)	6.92E-05					
Cigarettes	-132.89 (-169.15, -96.63)	1.67E-12					
Cigarettes: CBC	-33.83 (-43.05, -24.6)	1.63E-12					

Supplementary Table 9 This table presents instrumental variable regression results on cancer incidence rates.

Abbreviations:
MHY - Median household income

Supplementary Table 9 – ivreg, ipwtm, PC1Cancer (Continued)

Full Additive Model							
ivreg(PC1_Cancer ~ Cigarettes + AUD + PC1_Cannabinoids + Analgesics + Cocaine + Income + 6_Races)							
Caucasian	4.65 (3.96, 5.34)	<2.2E-16	0.7075	0.5569	105.6	9,740	<2.2E-16
African	0.37 (0.31, 0.43)	<2.2E-16					
PC1_Cannabinoid	0.35 (0.29, 0.41)	<2.2E-16					
Asian	14.83 (11.53, 18.13)	<2.2E-16					
Hispanic	0.31 (0.22, 0.39)	5.06E-12					
Cocaine	-9.62 (-18.72, -0.52)	0.0387					
AUD	-5.67 (-9.72, -1.63)	0.0061					
NHPI	-19.89 (-32.22, -7.55)	0.0016					
Analgesics	-0.51 (-0.78, -0.24)	0.0003					
Full Interactive Model							
ivreg(PC1_Cancer ~ Cigarettes * AUD * PC1_Cannabinoids + Analgesics + Cocaine + Income + 6_Races)							
African	0.4 (0.34, 0.46)	<2.2E-16	0.6789	0.5920	91.57	12,737	<2.2E-16
Caucasian	4.2 (3.52, 4.89)	<2.2E-16					
Asian	13.68 (10.26, 17.09)	1.54E-14					
Cigarettes: PC1_Cannabinoid	6.63 (4.8, 8.46)	2.76E-12					
Hispanic	0.21 (0.12, 0.31)	1.21E-05					
Cigarettes	7.56 (2.92, 12.21)	0.0015					
AUD	28.29 (8.85, 47.74)	0.0045					
Cigarettes: AUD: PC1_Cannabinoid	-20.76 (-38.11, -3.4)	0.0193					
NHPI	-18.65 (-31.1, -6.2)	0.0034					
Analgesics	-0.42 (-0.69, -0.14)	0.0030					
Cigarettes: AUD	-136.21 (-214.14, -58.27)	0.0007					
PC1_Cannabinoid	-0.8 (-1.1, -0.5)	2.41E-07					
Full Additive Model with Cannabinoids							
ivreg(PC1_Cancer ~ Cigarettes + THC + CBG + CBC + AUD + Analgesics + Cocaine + Income + 6_Races)							
African	0.33 (0.28, 0.39)	<2.2E-16	0.6749	0.5968	139.6	8,741	<2.2E-16
Caucasian	3.88 (3.21, 4.56)	<2.2E-16					
THC	1.48 (1.19, 1.77)	<2.2E-16					
Asian	13.64 (10.42, 16.86)	4.86E-16					
Hispanic	0.21 (0.12, 0.29)	2.49E-06					
Analgesics	-0.4 (-0.66, -0.13)	0.0032					
NHPI	-19.81 (-31.73, -7.88)	0.0012					
CBC	-0.85 (-1.24, -0.45)	2.68E-05					
Full Interactive Model with Cannabinoids							
ivreg(PC1_Cancer ~ Cigarettes * THC * CBG * CBC + AUD + Analgesics + Cocaine + Income + 6_Races)							
African	0.34 (0.29, 0.4)	<2.2E-16	0.6373	0.6404	75.12	18,731	<2.2E-16
Caucasian	3.38 (2.69, 4.07)	<2.2E-16					
Asian	13.09 (9.84, 16.34)	1.02E-14					
CBG	10.6 (4.39, 16.81)	0.0009					
THC: CBG: CBC	3.81 (1.54, 6.08)	0.0011					
Hispanic	0.16 (0.06, 0.25)	0.0011					
CBG: CBC	2.47 (0.93, 4)	0.0018					
THC: CBG	16.09 (5.82, 26.36)	0.0022					
CBC	7.52 (2.44, 12.59)	0.0038					
THC	46.13 (11.12, 81.15)	0.0100					
Cigarettes	2.38 (0.48, 4.29)	0.0144					
THC: CBC	11.15 (0.87, 21.42)	0.0339					
NHPI	-20.05 (-32.02, -8.08)	0.0011					
Cigarettes: THC: CBC	-75.46 (-120.13, -30.78)	0.0010					
Analgesics	-0.48 (-0.75, -0.21)	0.0006					
Cigarettes: THC	-313.69 (-471.53, -155.85)	0.0001					
Cigarettes: THC: CBG	-92.65 (-138.93, -46.37)	9.55E-05					
Cigarettes: THC: CBG: CBC	-21.6 (-31.98, -11.23)	4.97E-05					

Supplementary Table 10 – E-Values on Linear Models

Parameter	Estimate (C.I.)	E-Values
Quintile Comparisons		
Quintile 3 v 5		
PC1 Defects	2.51 (1.61, 3.42)	19.25, 11.25
PC1 ETOPFA	2.09 (1.25, 2.93)	12.90, 7.75
PC1 Cancers	0.54 (0.23, 0.85)	2.91, 2.63
Quintile 4 v 5		
PC1 Defects	1.85 (1.05, 2.65)	10.21, 6.24
PC1 ETOPFA	1.53 (0.77, 2.29)	7.52, 4.64
PC1 Cancers	0.16 (0.14, 0.47)	1.62, 1.38
Quintile 5 v Lower		
PC1 Defects	1.76 (1.22, 2.29)	9.57, 6.652
PC1 ETOPFA	1.59 (1.04, 2.14)	7.86, 5.43
PC1 Cancers	1.59 (1.35, 1.83)	2.68, 2.52
Legal Status		
Raw Data		
Decriminalized	0.62 (0.08, 1.16)	2.73, 1.34
Legal	2.57 (1.03, 4.11)	16.48, 4.16
ETOPFA Data		
Decriminalized	0.58 (0.04, 1.11)	2.61, 1.21
Legal	2.42 (0.87, 3.96)	14.35, 3.56
Cancer Data		
Medical	0.62 (0.44, 0.80)	2.99, 2.40
Decriminalized	0.52 (0.33, 0.70)	2.64, 2.09
Legal	0.69 (0.32, 1.05)	3.25, 2.05
Dichotomized Legal Status		
Illegal v Liberal		
PC1 Defects	0.46 (0.03, 0.89)	2.24, 1.58
PC1 ETOPFA	0.45 (0.03, 0.88)	2.23, 1.57
PC1 Cancers	0.58 (0.44, 0.72)	2.55, 2.43
Legal v Not_Legal		
PC1 Defects	2.43 (0.88, 3.98)	14.16, 5.86
PC1 ETOPFA	2.27 (0.72, 3.82)	12.42, 5.11
PC1 Cancers	0.45 (0.08, 0.83)	3.86, 3.44

Supplementary Table 10. This Table presents the results of E-Values from linear regression models.

Supplementary Table 11 – Minimum E-Value List

No.	Minimum E-Value
1	1.20E+18
2	2.14E+17
3	1.73E+07
4	3.53E+05
5	7.18E+04
6	1.14E+04
7	9.37E+03
8	1.25E+03
9	1.08E+03
10	109.21
11	105.18
12	65.62
13	47.55
14	44.16
15	22.14
16	17.54
17	12.01
18	11.25
19	9.91
20	9.79
21	9.43
22	7.75
23	7.75
24	7.00

No.	Minimum E-Value
25	6.65
26	6.57
27	6.24
28	5.86
29	5.43
30	5.11
31	4.64
32	4.16
33	3.56
34	3.44
35	2.99
36	2.63
37	2.57
38	2.52
39	2.43
40	2.40
41	2.25
42	2.23
43	2.17
44	2.12
45	2.09
46	2.05
47	1.94
48	1.93
49	1.87
50	1.76
51	1.58
52	1.57
53	1.53
54	1.38
55	1.34
56	1.21

Supplementary Table 11. This table presents a list of the minimum E-Values listed in descending order.

Supplementary Table 12: Cohen’s D & E- & P- Value’s for Legal Status Comparisons

	Illegal v Liberal	Legal v Not_Legal
**Cohen's D**		
PC1 Defects	0.40 (0.01, 0.79)	2.19 (0.75, 3.63)
PC1 ETOPFA	0.40 (0.01, 0.79)	2.05 (0.61, 3.49)
PC1 Cancers	0.60 (0.45, 0.75)	0.45 (0.07, 0.83)
**Effect Sizes**		
PC1 Defects	Small	Large
PC1 ETOPFA	Small	Large
PC1 Cancers	Medium	Small
**E-Values**		
PC1 Defects	2.24, 1.58	14.16, 5.86
PC1 ETOPFA	2.23, 1.57	12.42, 5.11
PC1 Cancers	2.55, 2.43	3.86, 3.44
**P-Levels of T-tests**		
PC1 Defects	0.0568	9.67E-43
PC1 ETOPFA	0.0563	3.71E-07
PC1 Cancers	2.66E-15	6.38E-04

Supplementary Table 12. This Table presents an analysis of effect sizes, Cohen’s D, P-values and E-values by legal status.

Supplementary Table 13 - ETOPFA Rates

Defect	QLD 2008	QLD 2009	QLD 2010	QLD Mean	Australia 2002-03	Australian ETOPFA Rate	US.Rate	USA.ETOPFA.Relative.Rate	Composite
Trisomy 21 (Down syndrome)	0.5	0.53	0.47	0.5	0.6	0.25	0.06	0.94	0.70
Trisomy 18	0.71	0.56	0.5	0.59	0.89	0.9	0.43	0.57	0.89
Trisomy 13	0.78	0.86	0.8	0.81	0.9	1.00	0.38	0.62	0.90
Turner syndrome					0.85	0.68			0.80
Deletion 22q11.2									0.90

Supplementary Table 13. This table presents applicable ETOPFA rates taken from References 64-66.

Supplementary Table 14 - Historical Sequence TOP Rates WA

Year	T21 Live Bihs	T21 ETOPFA	Total T21	WA.T21.TOP Rate	TOP_Rate	FMaxTR
1980	1.0987	0.0000	1.0987	0.0000	0.0000	0.0000
1981	0.8537	0.1254	0.9791	0.1275	0.1275	0.1645
1982	1.1136	0.1184	1.2320	0.0941	0.0941	0.1215
1983	1.0850	0.1115	1.1965	0.0923	0.0923	0.1191
1984	0.9987	0.1910	1.1897	0.1581	0.1581	0.2041
1985	1.1504	0.3283	1.4787	0.2183	0.2183	0.2818
1986	1.1651	0.4079	1.5729	0.2496	0.2496	0.3222
1987	1.0283	0.3144	1.3427	0.2131	0.2131	0.2750
1988	1.0863	0.5670	1.6533	0.3323	0.3323	0.4289
1989	0.9783	0.2932	1.2716	0.2276	0.2276	0.2938
1990	1.1156	0.3368	1.4523	0.2135	0.2135	0.2756
1991	1.0437	0.4235	1.4673	0.2722	0.2722	0.3514
1992	1.2603	0.5247	1.7851	0.2834	0.2834	0.3658
1993	1.4625	0.5250	1.9875	0.2521	0.2521	0.3253
1994	0.6695	0.8570	1.5265	0.5557	0.5557	0.7173
1995	1.0231	1.1168	2.1399	0.5080	0.5080	0.6557
1996	1.0882	0.8358	1.9241	0.4141	0.4141	0.5345
1997	0.8433	1.0092	1.8525	0.5322	0.5322	0.6869
1998	0.7498	0.9517	1.7015	0.5301	0.5301	0.6842
1999	0.8149	1.0241	1.8390	0.5399	0.5399	0.6969
2000	1.2767	1.7022	2.9789	0.5606	0.5606	0.7236
2001	0.7289	1.3779	2.1068	0.6454	0.6454	0.8330
2002	1.1402	1.5080	2.6482	0.5430	0.5430	0.7008
2003	0.8232	1.4073	2.2304	0.6058	0.6058	0.7819
2004	1.0974	1.7032	2.8006	0.5800	0.5800	0.7487
2005	1.0400	1.8621	2.9021	0.6341	0.6341	0.8184
2006	0.8383	1.6893	2.5276	0.6539	0.6539	0.8440
2007	1.0044	1.6823	2.6868	0.6008	0.6008	0.7755
2008	0.7523	1.5095	2.2618	0.6224	0.6224	0.8033
2009	0.9761	1.9857	2.9618	0.6489	0.6489	0.8375
2010	0.9582	2.2352	3.1935	0.6997	0.6997	0.9031
2011	0.6527	2.2291	2.8819	0.7815	0.7815	1.0087
2012	0.7383	2.1802	2.9185	0.7531	0.7531	0.9720
2013	0.7200	2.0825	2.8024	0.7461	0.7461	0.9630
2014	0.5122	1.8075	2.3198	0.7748	0.7748	1.0000
2015	0.5122	1.8075	2.3198	0.7748	0.7748	1.0000

Supplementary Table 14. This table presents the longitudinal time series of the regression of ETOPFA rates in the Western Australian Down syndrome series (Reference 143).

Supplementary Table 15 - Quintiles Composition

State	Quintile 1	Quintile 2	Quintile 3	Quintile 4	Quintile 5
Alabama	14	1	0	0	0
Alaska	0	0	0	0	15
Arizona	0	3	7	5	0
Arkansas	0	6	9	0	0
California	0	0	0	15	0
Colorado	0	0	0	1	14
Connecticut	0	0	2	12	1
Delaware	0	0	6	9	0
Florida	0	0	14	1	0
Georgia	2	3	8	2	0
Hawaii	0	1	4	8	2
Idaho	6	9	0	0	0
Illinois	0	2	13	0	0
Indiana	0	1	2	12	0
Iowa	14	1	0	0	0
Kansas	4	11	0	0	0
Kentucky	0	7	7	1	0
Louisiana	7	8	0	0	0
Maine	0	0	0	0	15
Maryland	2	3	1	9	0
Massachusetts	0	0	0	3	12
Michigan	0	0	0	15	0
Minnesota	0	3	7	4	1
Mississippi	14	1	0	0	0
Missouri	0	0	14	1	0
Montana	0	0	0	0	15
Nebraska	9	4	2	0	0
Nevada	0	0	4	9	2
New Hampshire	0	0	0	0	15
New Jersey	8	7	0	0	0
New Mexico	0	0	0	15	0
New York	0	0	0	15	0
Noh Carolina	0	11	4	0	0
Noh Dakota	15	0	0	0	0
Ohio	0	0	13	2	0
Oklahoma	10	4	1	0	0
Oregon	0	0	0	0	15
Pennsylvania	0	2	12	1	0
Rhode Island	0	0	0	0	15
South Carolina	4	6	5	0	0
South Dakota	7	7	1	0	0
Tennessee	1	8	4	2	0
Texas	15	0	0	0	0
Utah	15	0	0	0	0
Vermont	0	0	0	0	15
Virginia	2	8	4	1	0
Washington	0	0	0	2	13
West Virginia	1	12	2	0	0
Wisconsin	0	14	1	0	0
Wyoming	6	6	2	1	0

Supplementary Table 15. This table illustrates the quintile membership of each state by the aggregate of years for which they were in each quintile.

Supplementary Figures

Supplementary Figure List

Figure	Subject
Supplementary Figure 1	Scatterplot Matrix of CAR's by Substance Exposure
Supplementary Figure 2	CAR's and Cancers by Cannabis Exposure
Supplementary Figure 3	CAR's and Cancers by THC Exposure
Supplementary Figure 4	CAR's and Cancers by Cannabinol Exposure
Supplementary Figure 5	CAR's and Cancers by Cannabichromene Exposure
Supplementary Figure 6	CAR's and Cancers by Cannabigerol Exposure
Supplementary Figure 7	ETOPFACAR's and Cancers by Cannabis Exposure
Supplementary Figure 8	ETOPFACAR's and Cancers by Cannabinol Exposure
Supplementary Figure 9	ETOPFACAR's and Cancers by Cannabichromene Exposure
Supplementary Figure 10	ETOPFACAR's and Cancers by Cannabigerol Exposure
Supplementary Figure 11	Corrplot Correlogram of CAR's, Cancers and Substances - Pearson Correlation Coefficients
Supplementary Figure 12	Corrplot Correlogram of CAR's, Cancers and Substances - P-Values - Significance Levels
Supplementary Figure 13	Quintile Analysis - CAR Data
Supplementary Figure 14	Quintile Analysis - ETOPFACAR Data
Supplementary Figure 15	Quintile Analysis - Cancer Data
Supplementary Figure 16	Effect of Legal Status on CAR Data
Supplementary Figure 17	Effect of Legal Status on ETOPFACAR Data
Supplementary Figure 18	Effect of Legal Status on Cancer Data

Supplementary Figure 1 Chromsomal Anomalies by Substance Exposure - CAR Data

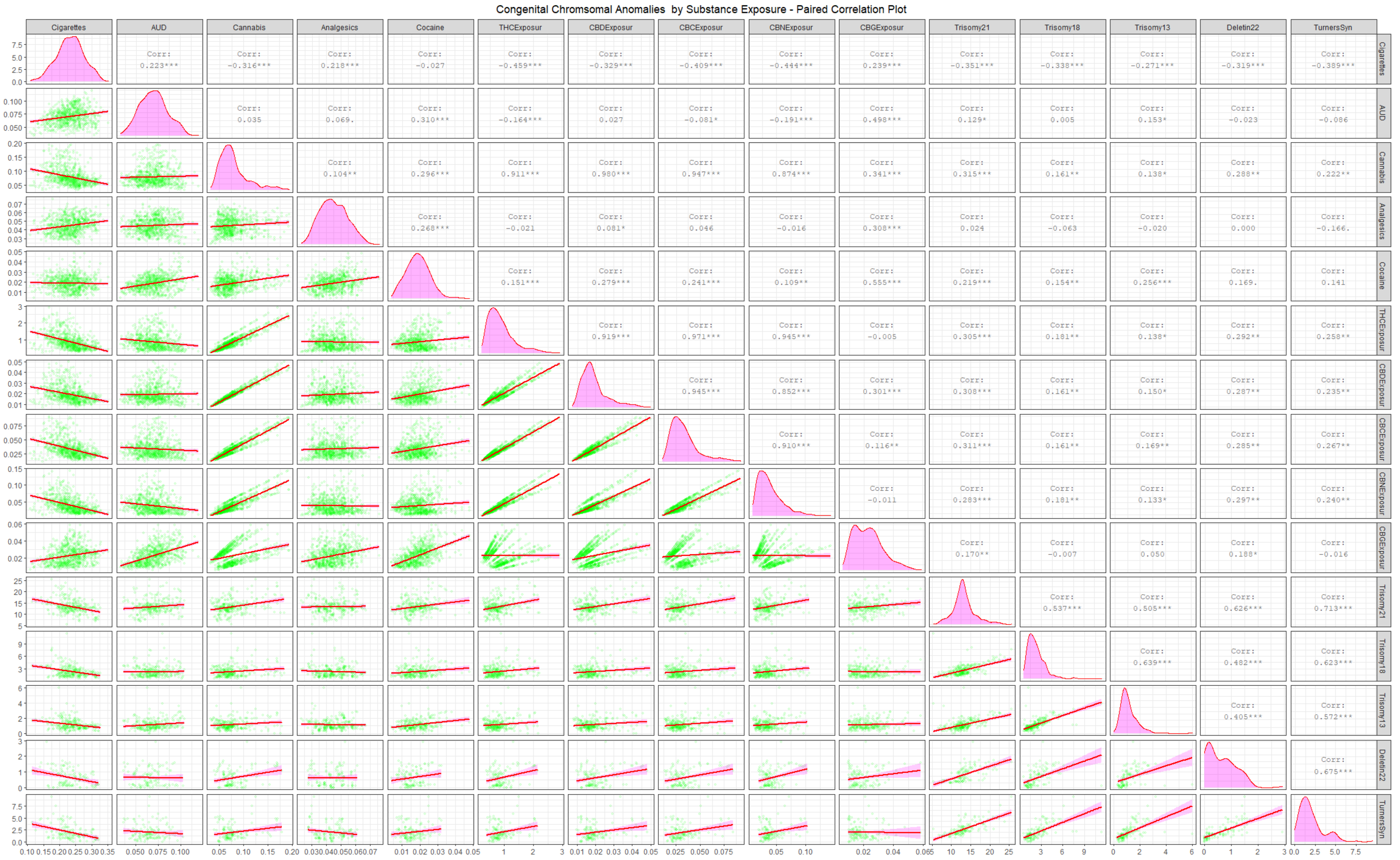

Caption to Supplementary Figure 1

Supplementary Figure 1.: Paired scatterplot matrix of CAR's against substance and cannabinoid exposures.

Supplementary Figure 2 - CAR Data ~ Cannabis

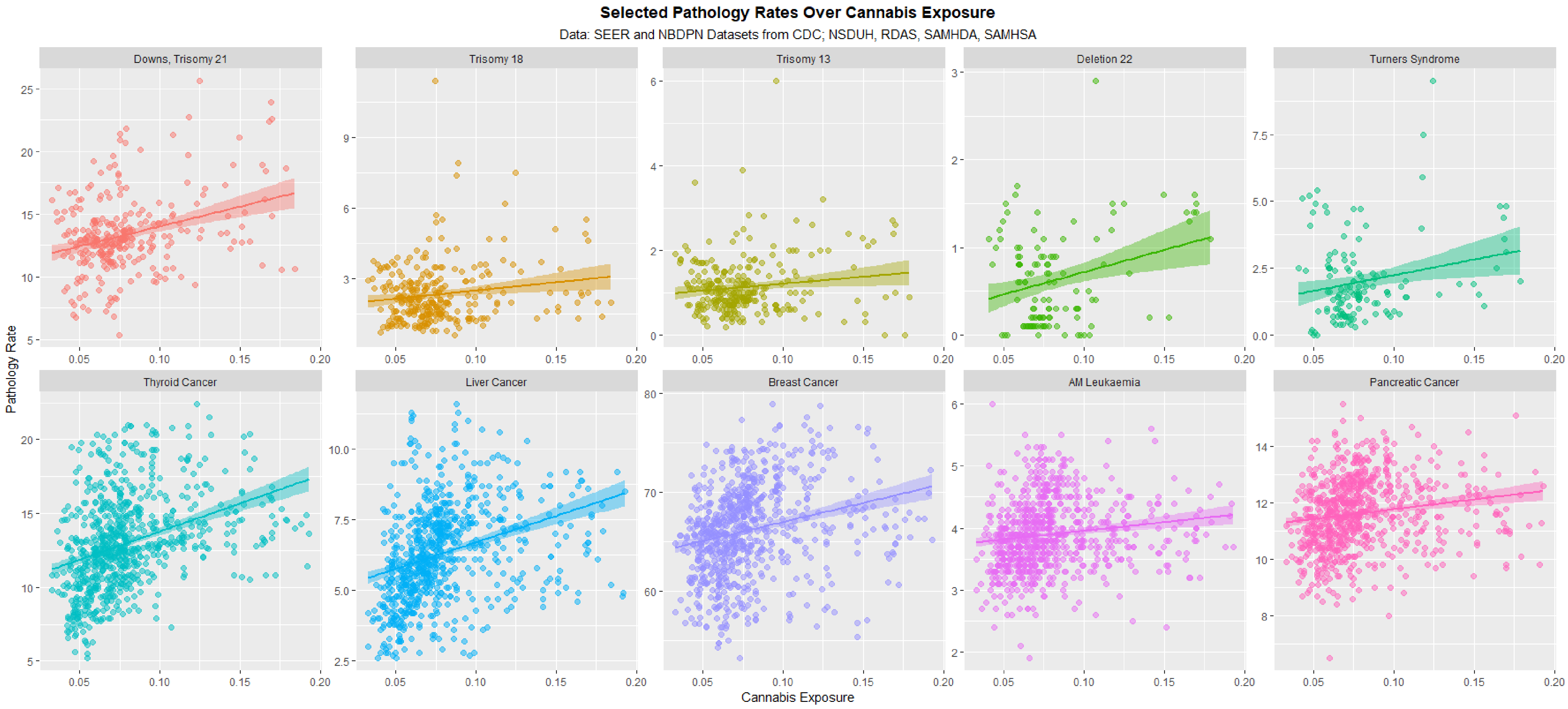

Supplementary Figure 2.: Scatterplots of pathologies of interest as a function of cannabis exposure for CAR Data.

Supplementary Figure 3 - CAR Data ~THC

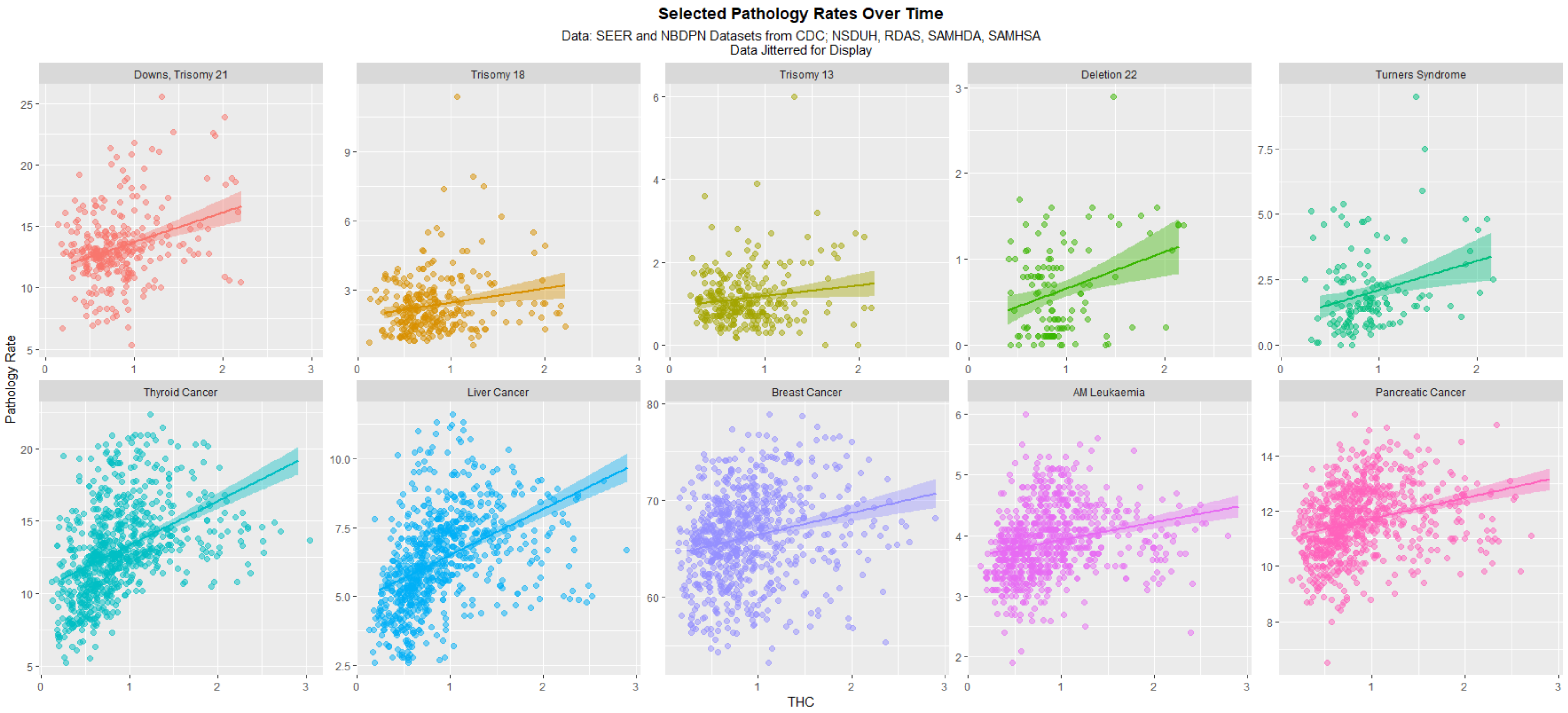

Supplementary Figure 3.: Scatterplots of pathologies of interest as a function of THC exposure for CAR Data.

Supplementary Figure 4 - CAR Data ~ Cannabinol

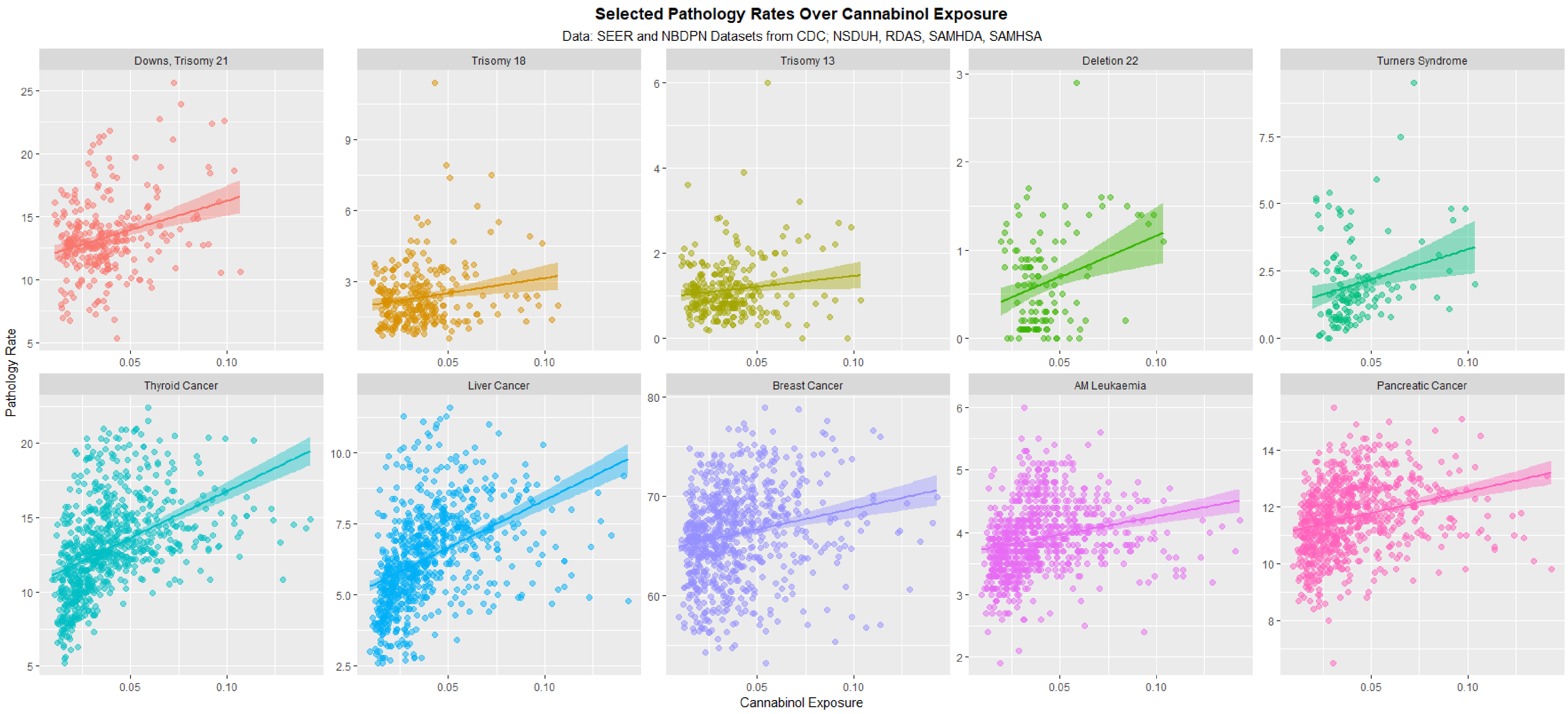

Supplementary Figure 4.: Scatterplots of pathologies of interest as a function of cannabinol exposure for CAR Data.

Supplementary Figure 5 - CAR Data ~ Cannabichromene

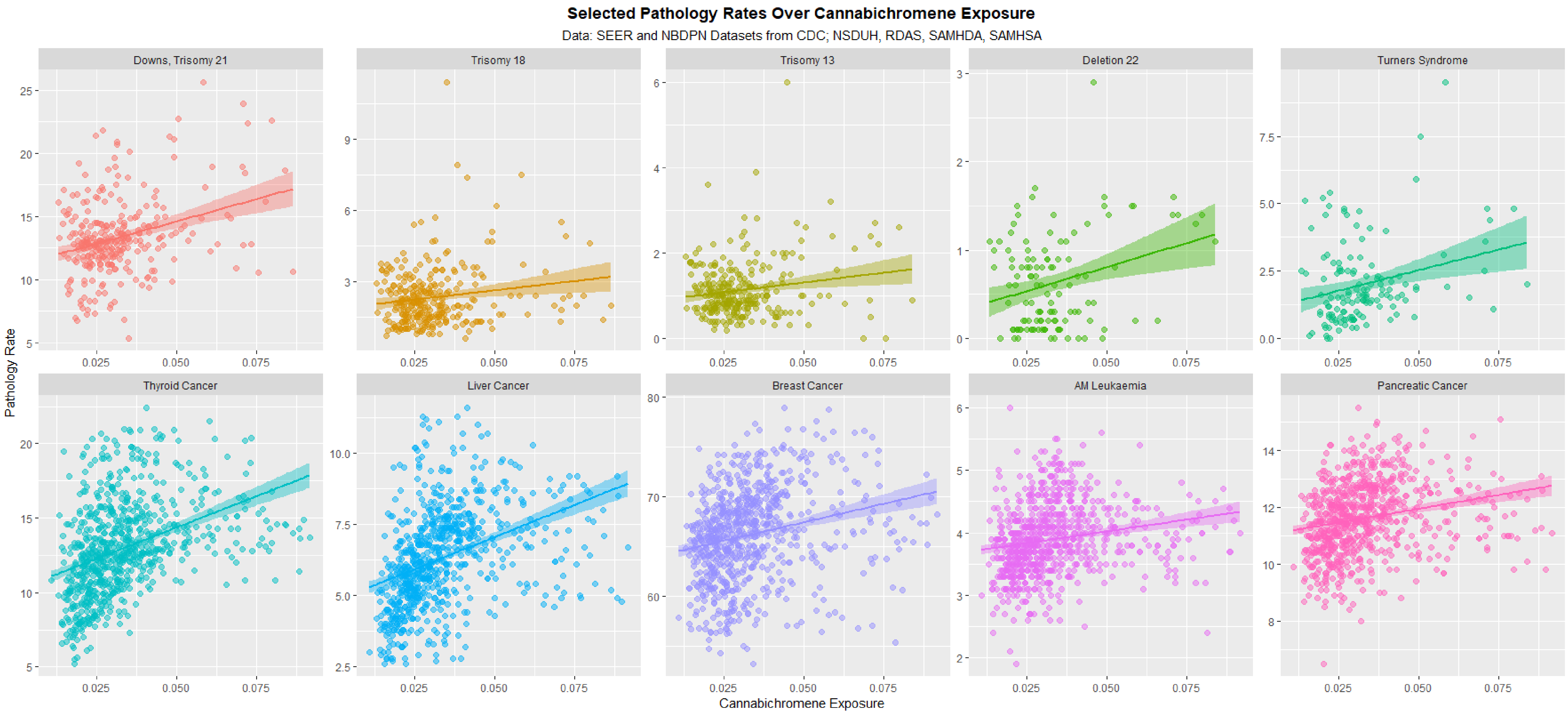

Supplementary Figure 5.: Scatterplots of pathologies of interest as a function of cannabichromene exposure for CAR Data.

Supplementary Figure 6 - CAR Data ~ Cannabigerol

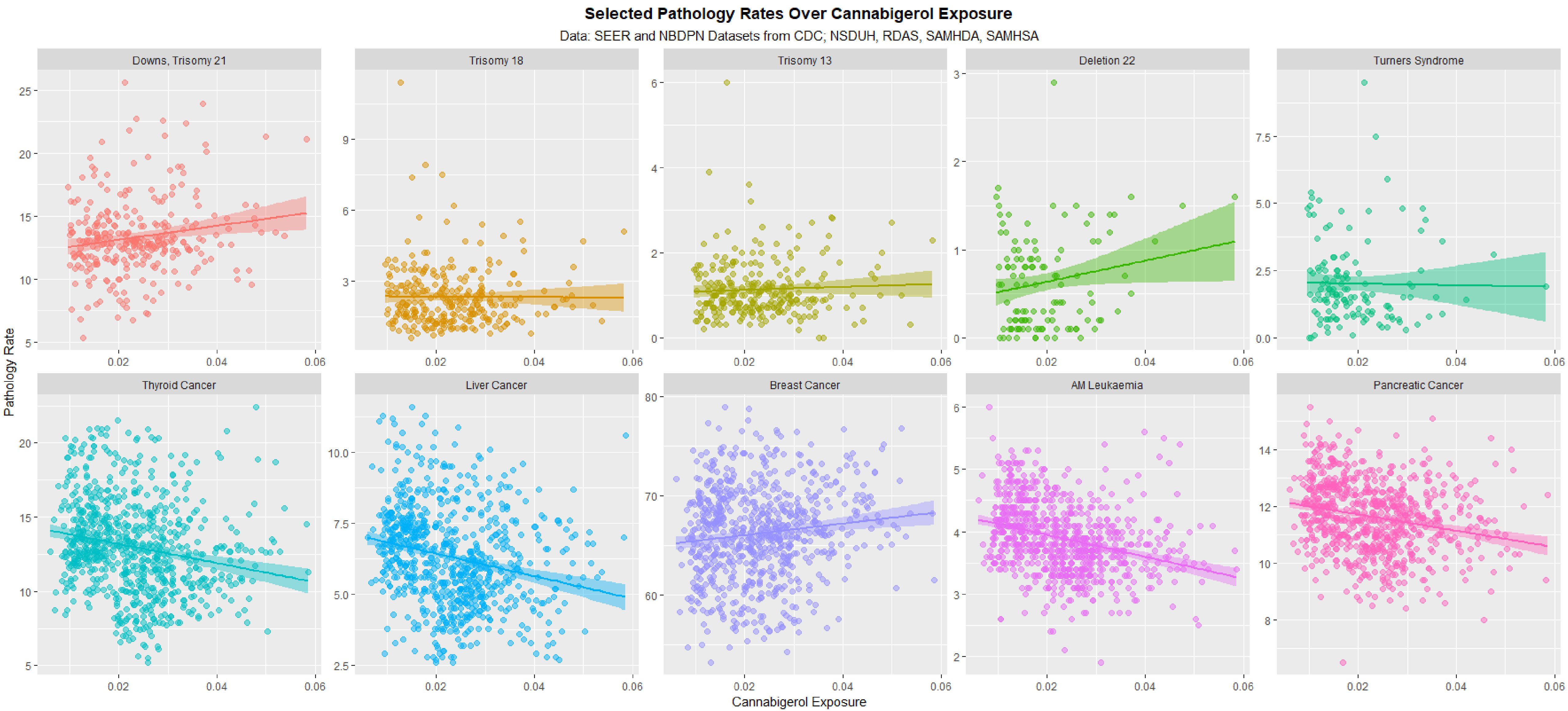

Supplementary Figure 6.: Scatterplots of pathologies of interest as a function of cannabigerol exposure for CAR Data.

Supplementary Figure 7 - ETOPFACAR Data ~ Cannabis

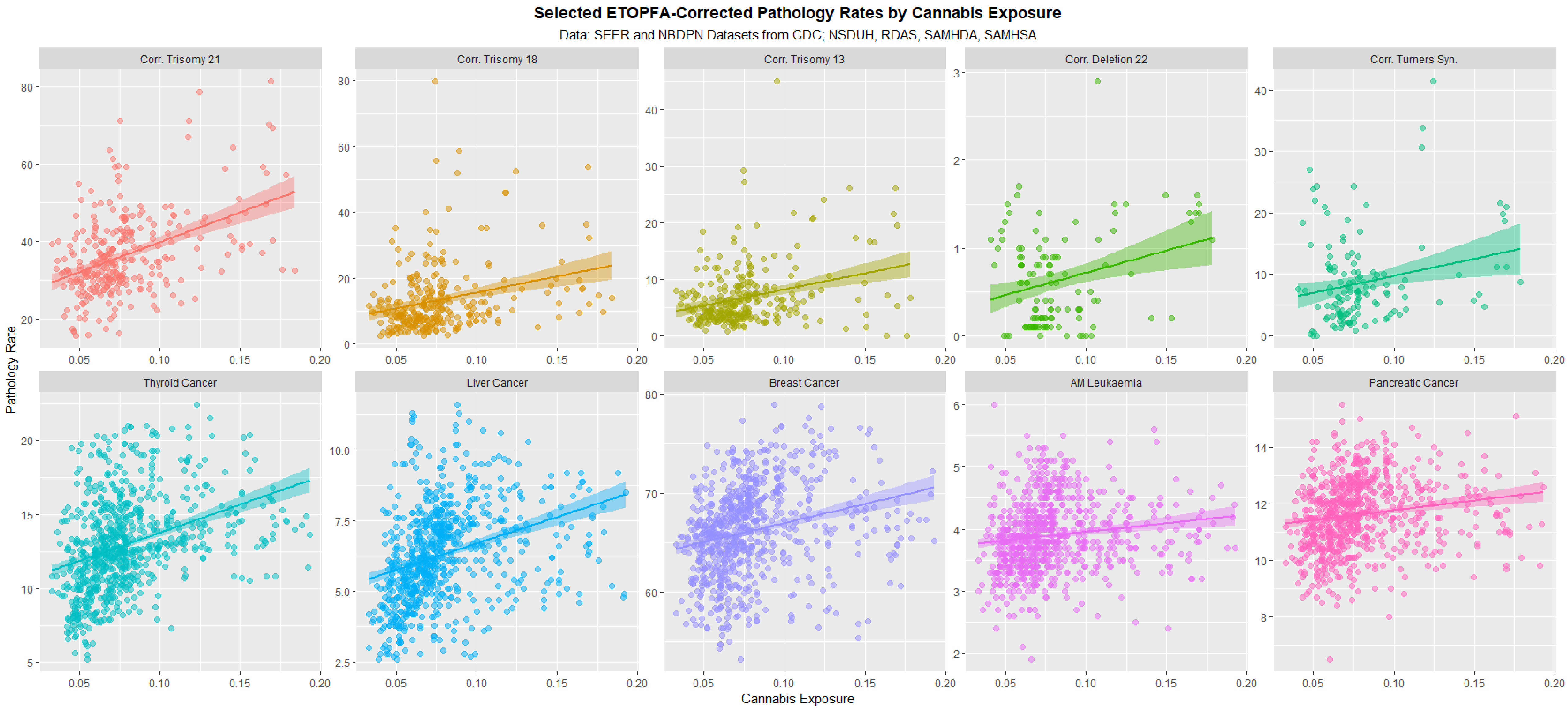

Supplementary Figure 7.: Scatterplots of pathologies of interest as a function of cannabis exposure for ETOPFACAR Data.

Supplementary Figure 8 - ETOPFACAR Data ~ Cannabinol

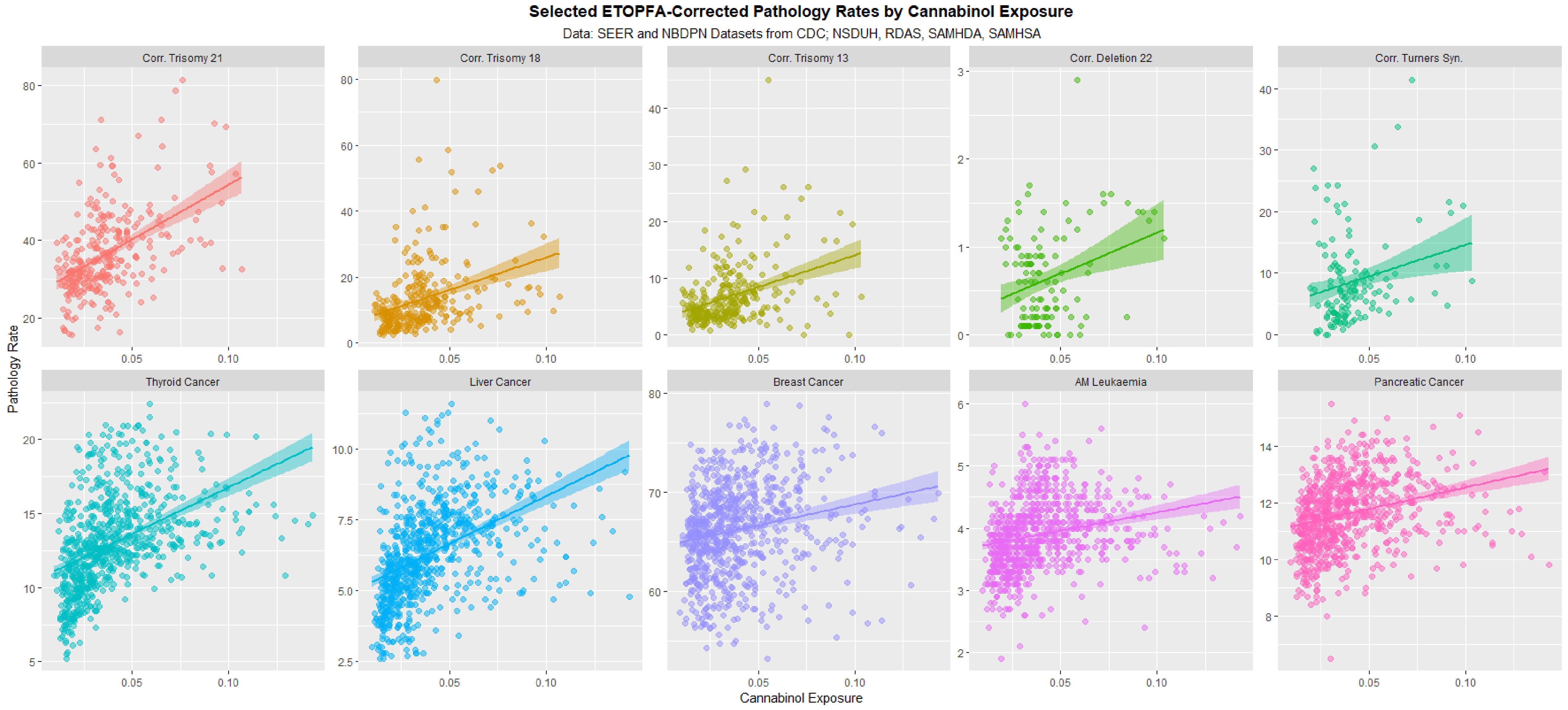

Supplementary Figure 8.: Scatterplots of pathologies of interest as a function of cannabinol exposure for ETOPFACAR Data.

Supplementary Figure 9 - ETOPFACAR Data ~ Cannabichromene

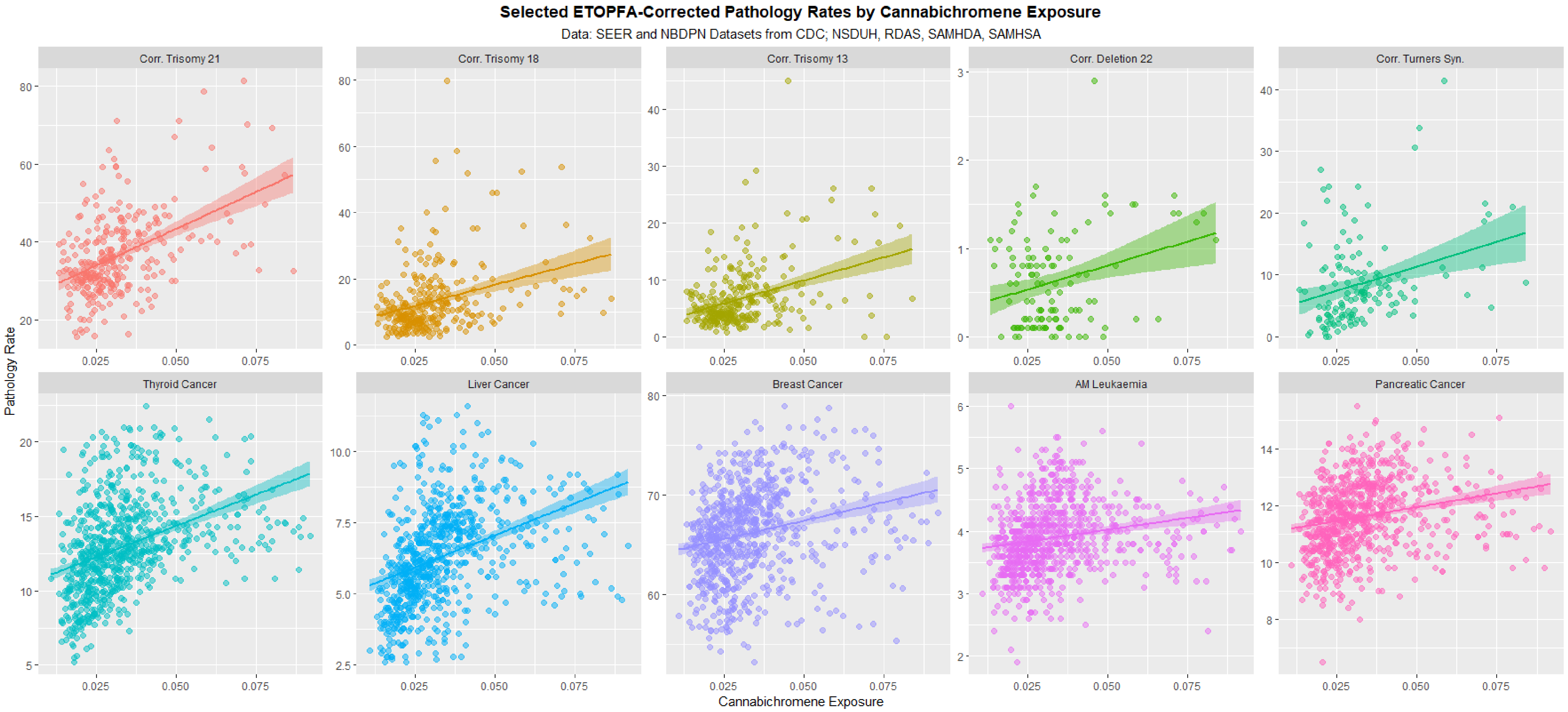

Supplementary Figure 9.: Scatterplots of pathologies of interest as a function of cannabichromene exposure for ETOPFACAR Data.

Supplementary Figure 10 - ETOPFACAR Data ~ Cannabigerol

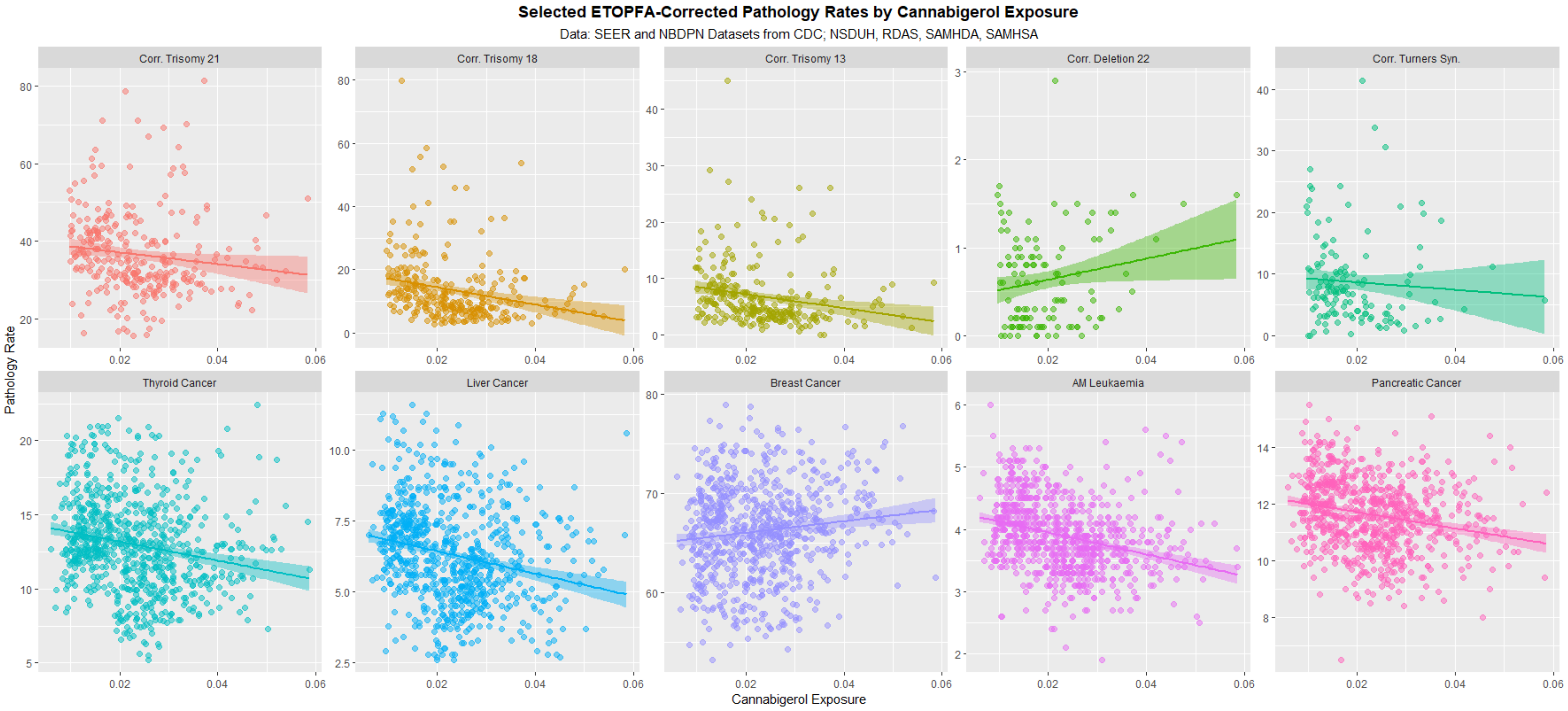

Supplementary Figure 10.: Scatterplots of pathologies of interest as a function of cannabigerol exposure for ETOPFACAR Data.

Supplementary Figure 11 – Corrplot Correlogram CAR Covariates – Pearson Correlation Coefficients

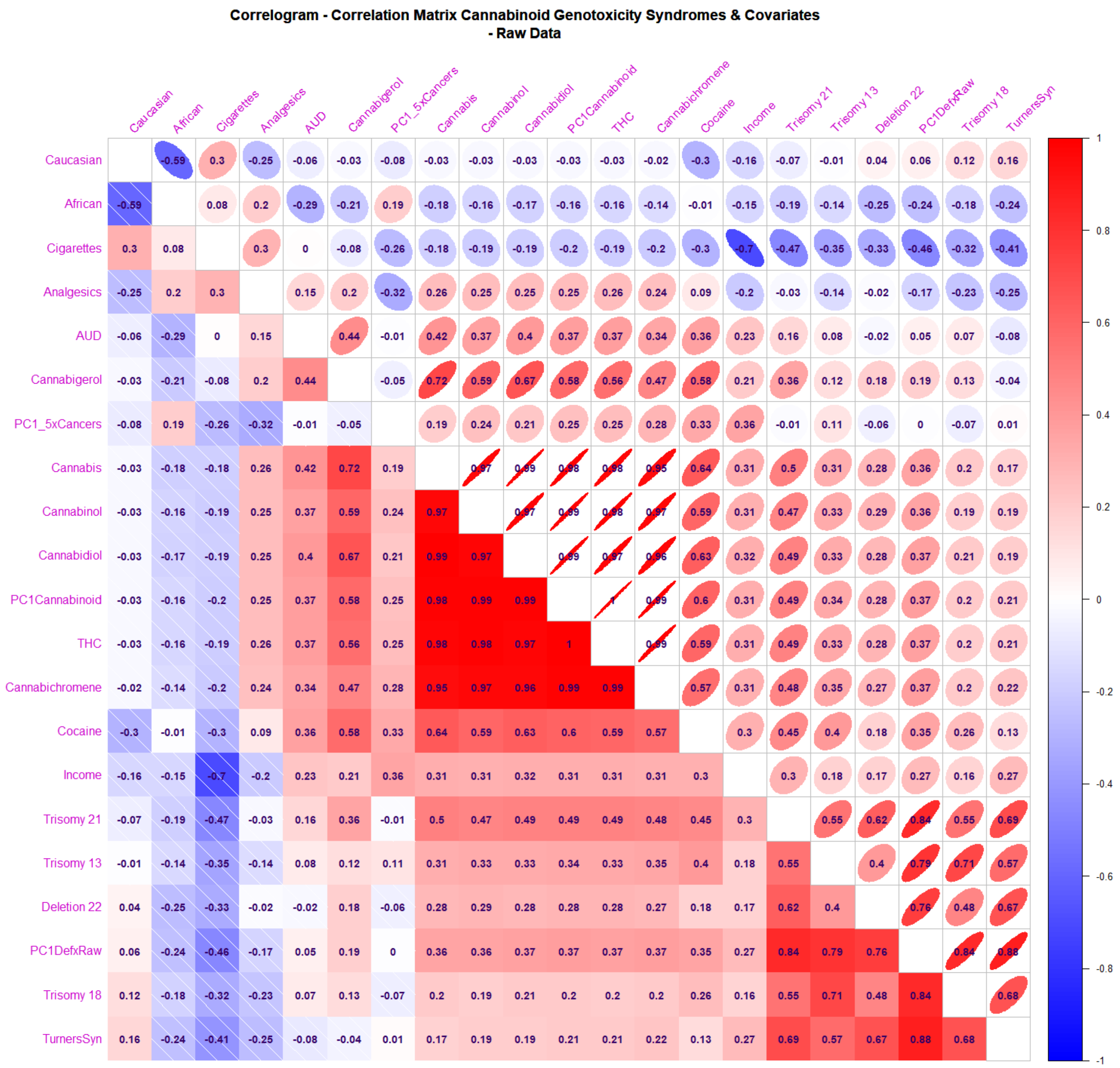

Caption:

Supplementary Figure 11.: Corrplot correlogram of the Pearson correlation coefficients for the relationship between CAR's and substance and cannabinoid exposure.

Supplementary Figure 12 – Corrplot Correlogram CAR Covariates - Significance Levels as P-Values

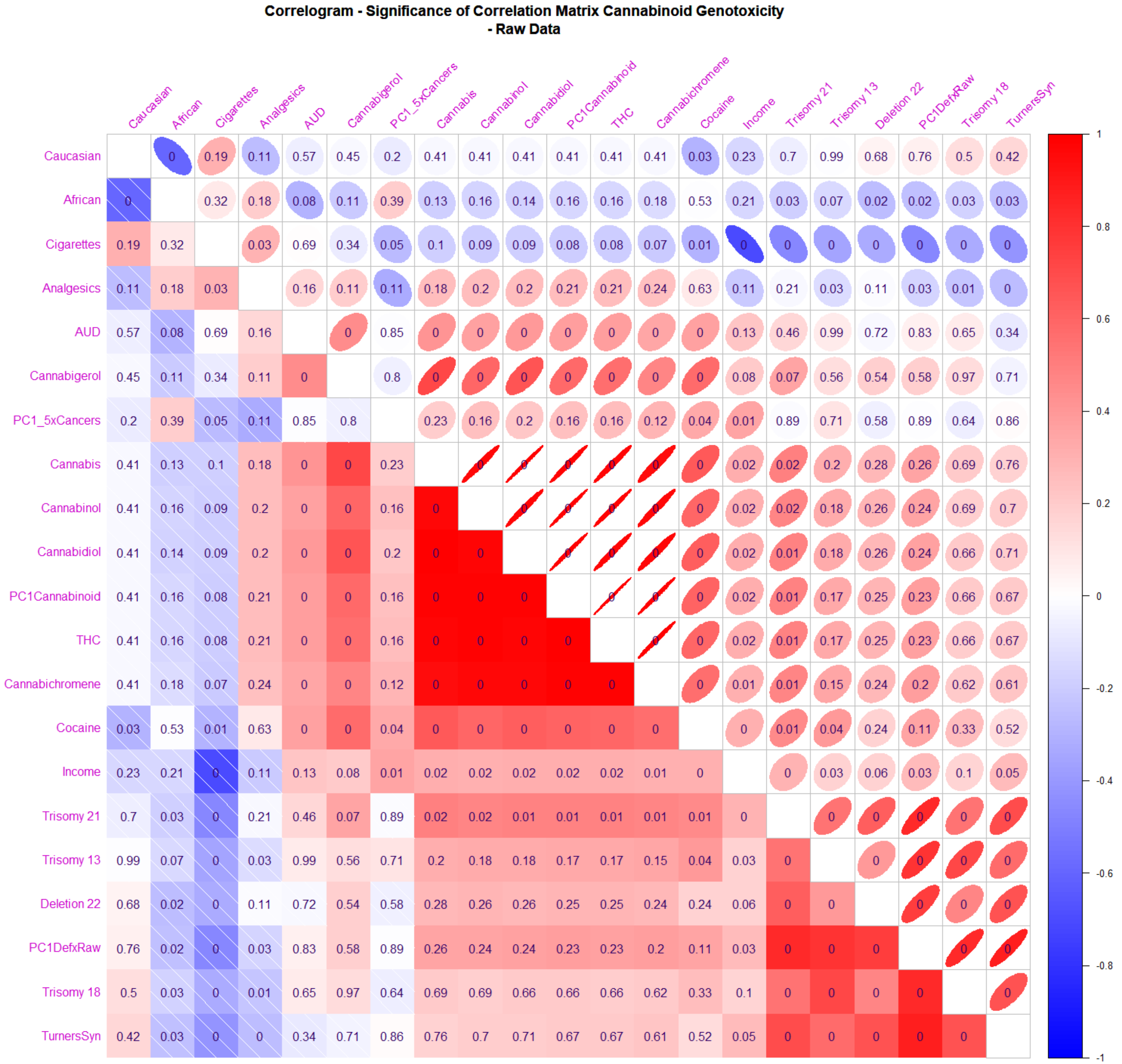

Caption:
Supplementary Figure 12.: Corrplot correlogram of the significance of the Pearson correlation coefficients for the relationship between CAR's and substance and cannabinoid exposure.

Supplementary Figure 13 Quintiles – CAR Data

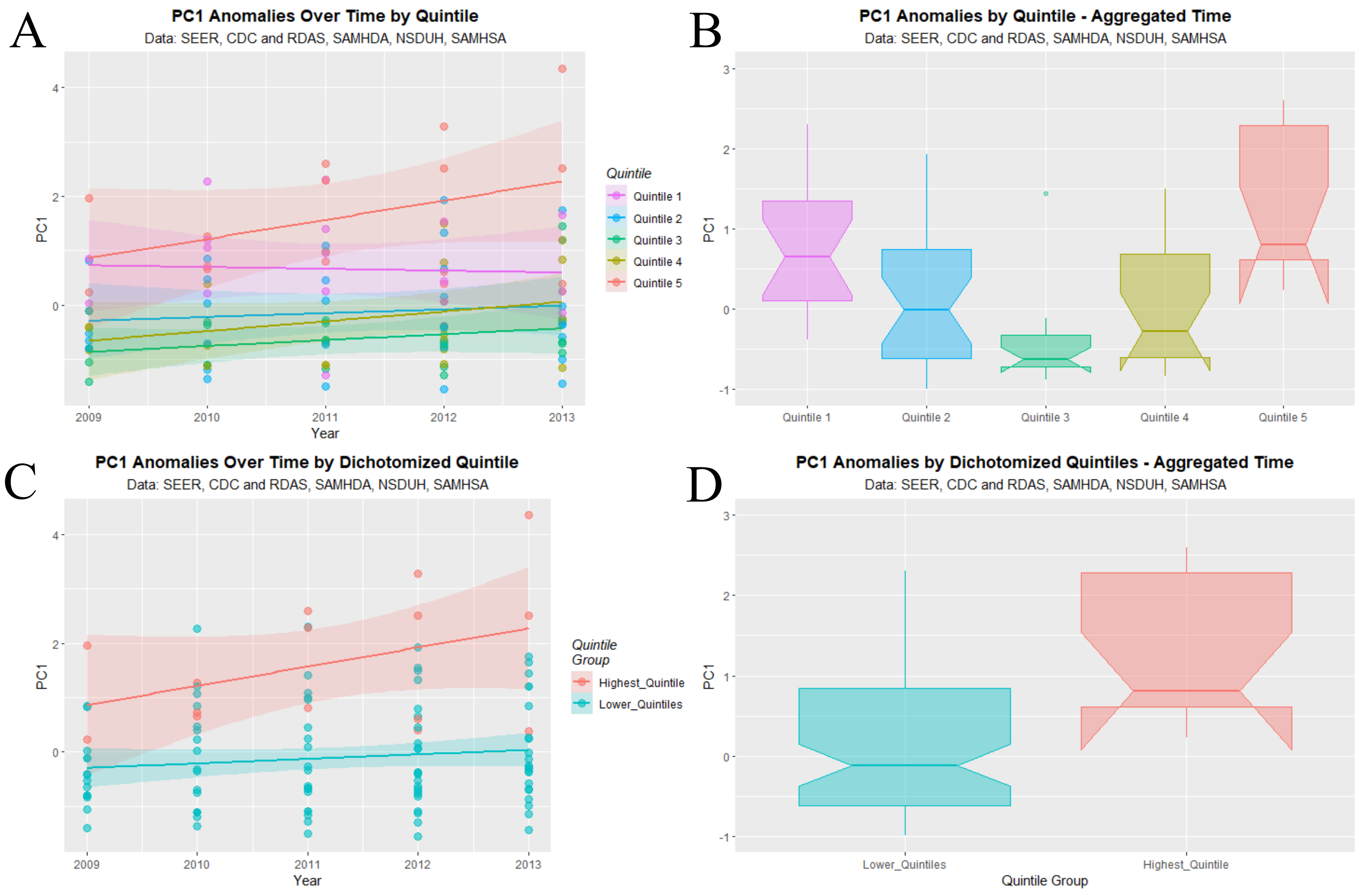

Supplementary Figure 13.: Effect of Cannabis exposure quintiles on PC1 for CAR data. (A) scatterplot over time for PC1 for CAR's. (B) Boxplot for PC1 CAR time-aggregated data by cannabis exposure quintiles as in (A). (C) Scatterplot over time for PC1 for CAR data dichotomized as the highest quintile v the lower four quintiles. (D) Boxplot for PC1 for CAR data dichotomized as the highest quintile v the lower four quintiles. (Created in R-Studio version 1.3.1093 using ggplot version 3.3.2).

Supplementary Figure 14 Quintiles – ETOPFA Data

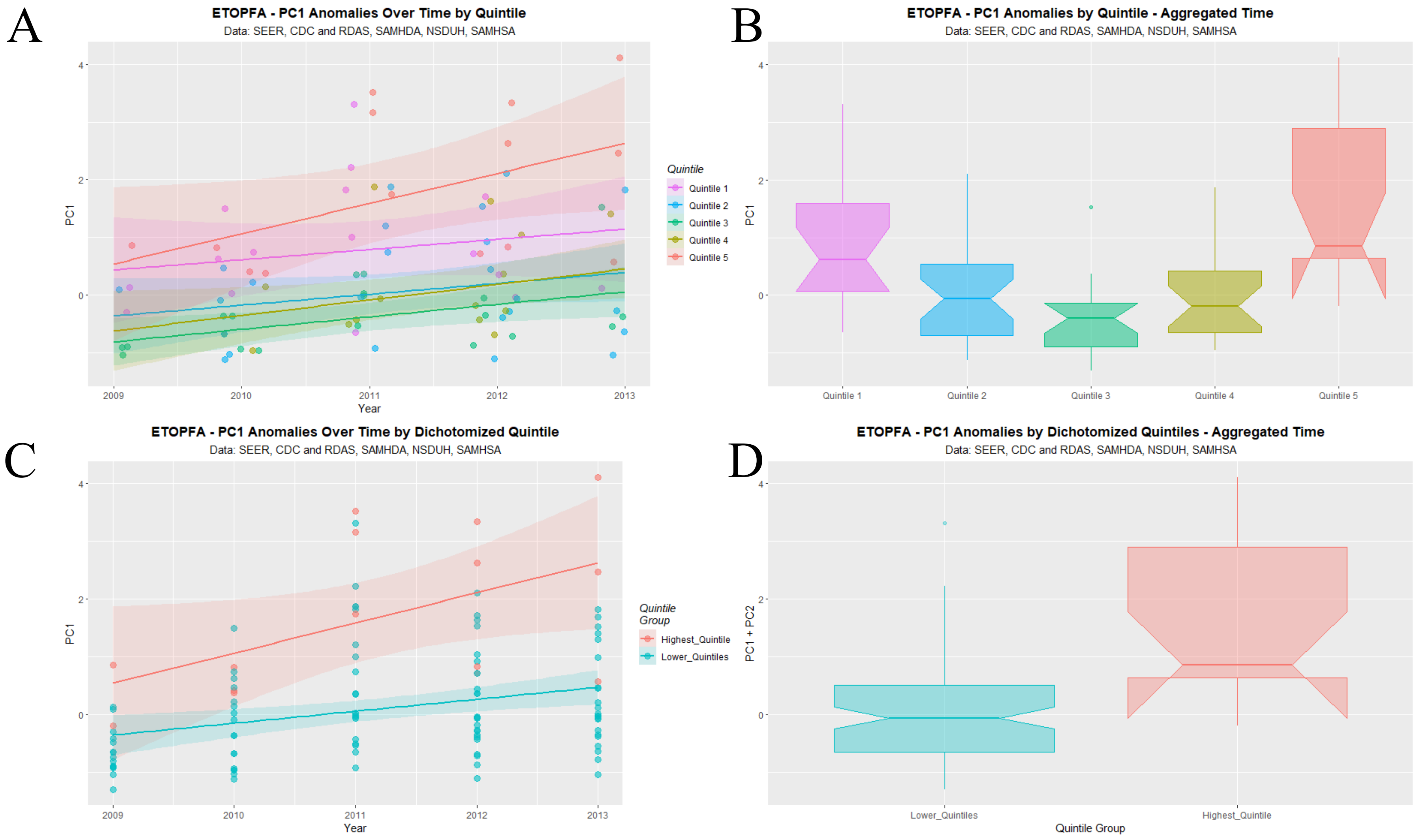

Supplementary Figure 14.: Effect of Cannabis exposure quintiles on PC1 for ETOPFACAR data. (A) scatterplot over time for PC1 for ETOPFACAR's. (B) Boxplot for PC1 ETOPFACAR time-aggregated data by cannabis exposure quintiles as in (A). (C) Scatterplot over time for PC1 for ETOPFACAR data dichotomized as the highest quintile v the lower four quintiles. (D) Boxplot for PC1 for ETOPFACAR data dichotomized as the highest quintile v the lower four quintiles. (Created in R-Studio version 1.3.1093 using ggplot version 3.3.2).

Supplementary Figure 15 Quintiles – Cancers

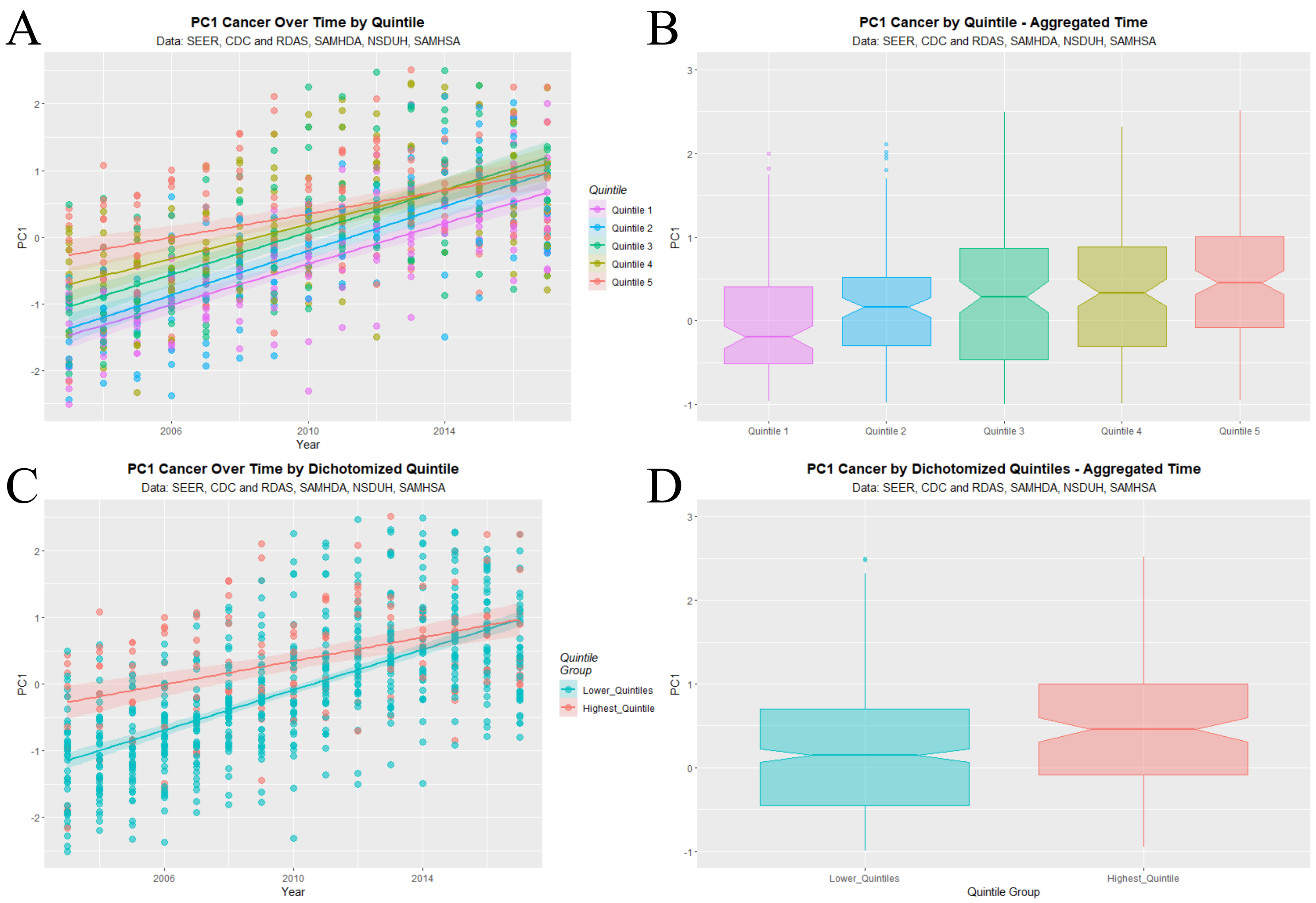

Supplementary Figure 15.: Effect of Cannabis exposure quintiles on PC1 for cancer data. (A) scatterplot over time for PC1 for cancers. (B) Boxplot for PC1 cancer time-aggregated data by cannabis exposure quintiles as in (A). (C) Scatterplot over time for PC1 for cancer data dichotomized as the highest quintile v the lower four quintiles. (D) Boxplot for PC1 for cancer data dichotomized as the highest quintile v the lower four quintiles. (Created in R-Studio version 1.3.1093 using ggplot version 3.3.2).

Supplementary Figure 16 – Legal Status – CAR Data

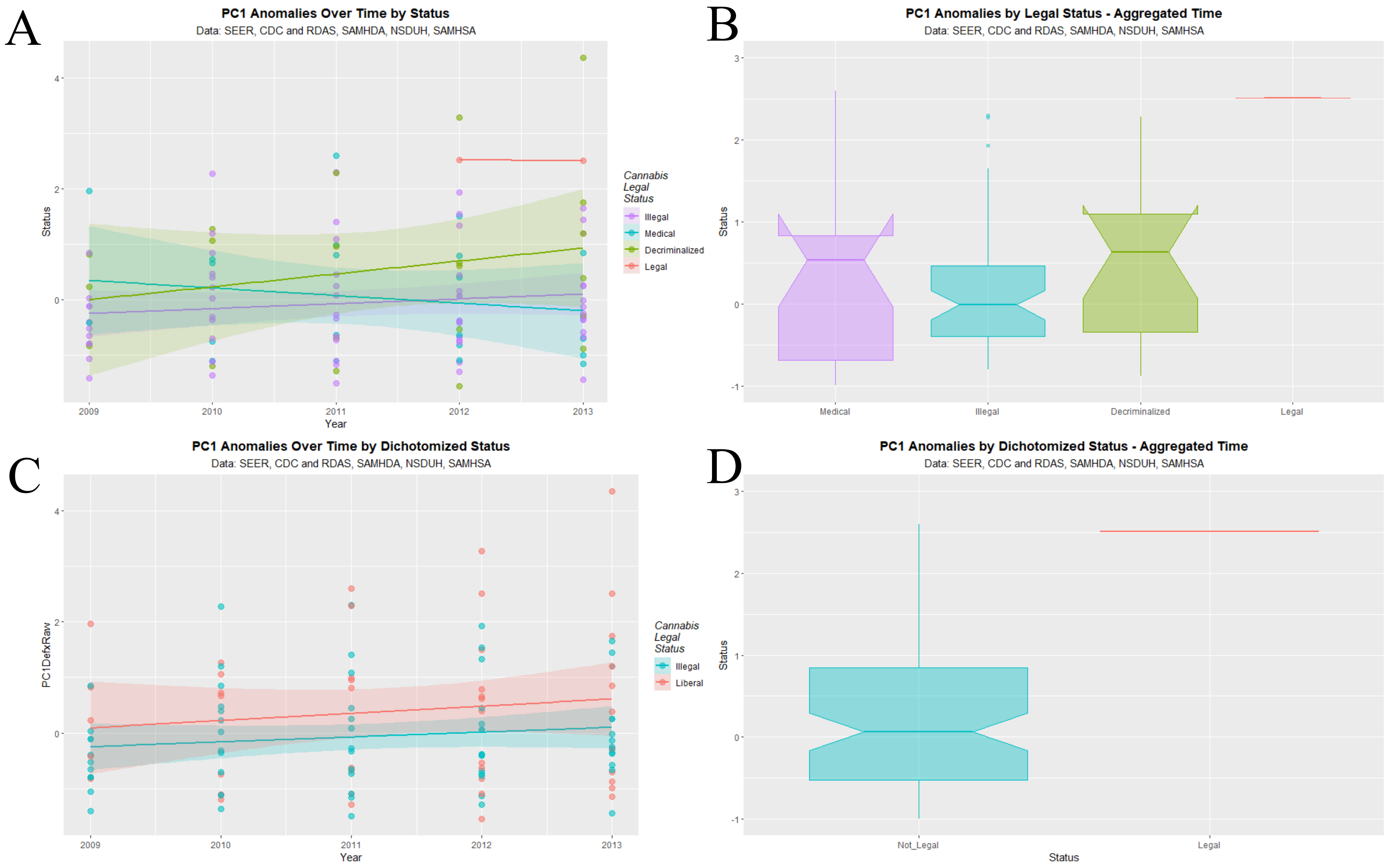

Supplementary Figure 16.: Effect of Cannabis legal status on PC1 for CAR data. (A) scatterplot over time for PC1 for CAR's for legal status. (B) Boxplot for PC1 for CAR incidence by legal status with aggregated time. (C) Scatterplot over time for PC1 for CAR incidence aggregated over time dichotomized as liberal v. illegal regimes. (D) Boxplot for PC1 for CAR incidence of dichotomized legal status over aggregated time dichotomized as in (C). (Created in R-Studio version 1.3.1093 using ggplot version 3.3.2).

Supplementary Figure 17 - Legal Status – ETOPFACAR Data

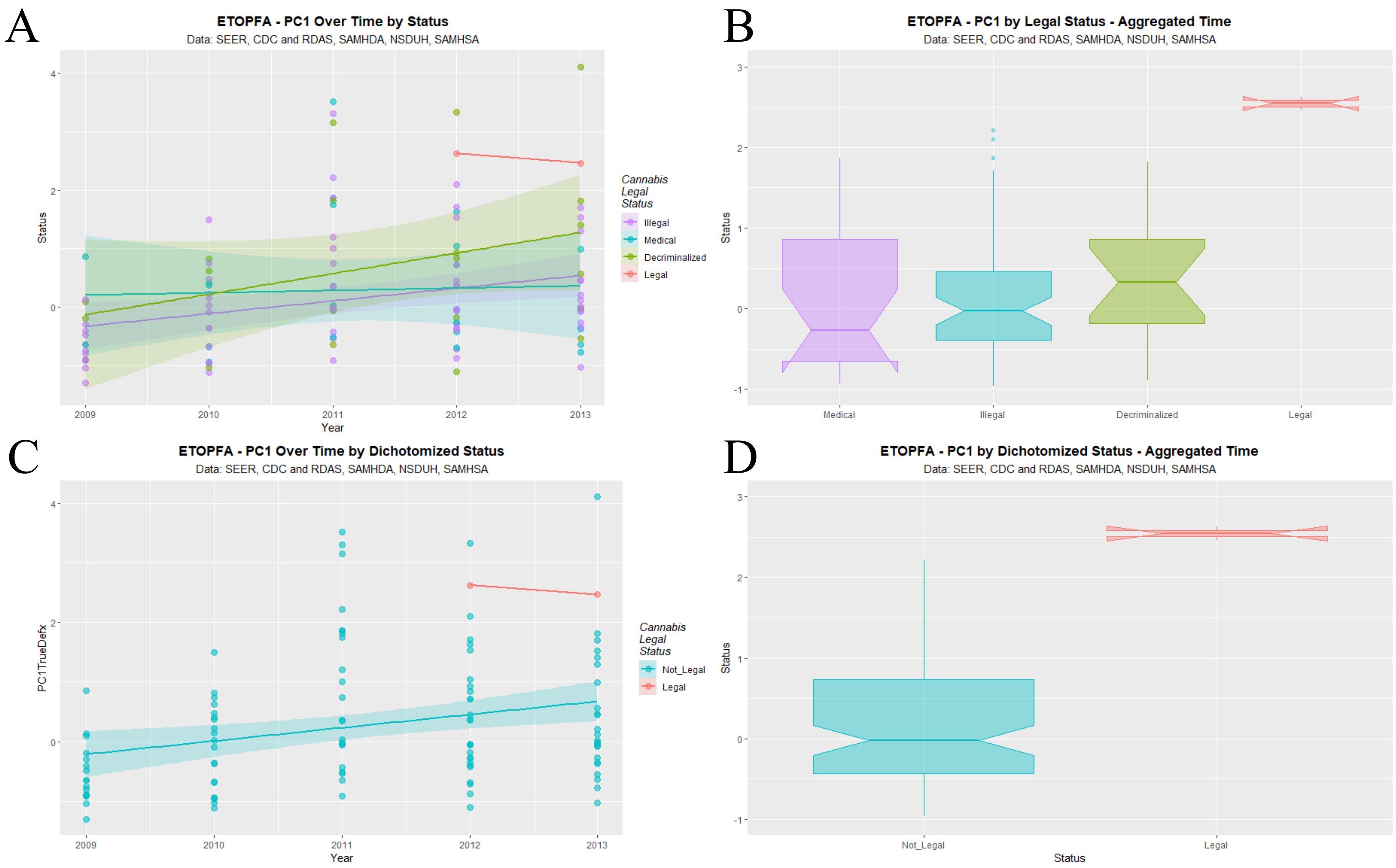

Supplementary Figure 17.: Effect of Cannabis legal status on PC1 for ETOPFACAR data. (A) scatterplot over time for PC1 for ETOPFACAR's for legal status. (B) Boxplot for PC1 for ETOPFACAR incidence by legal status with aggregated time. (C) Scatterplot over time for PC1 for ETOPFACAR incidence aggregated over time dichotomized as liberal v. illegal regimes. (D) Boxplot for PC1 for ETOPFACAR incidence of dichotomized legal status over aggregated time dichotomized as in (C). (Created in R-Studio version 1.3.1093 using ggplot version 3.3.2).

Supplementary Figure 18 - Legal Status – Cancer Data

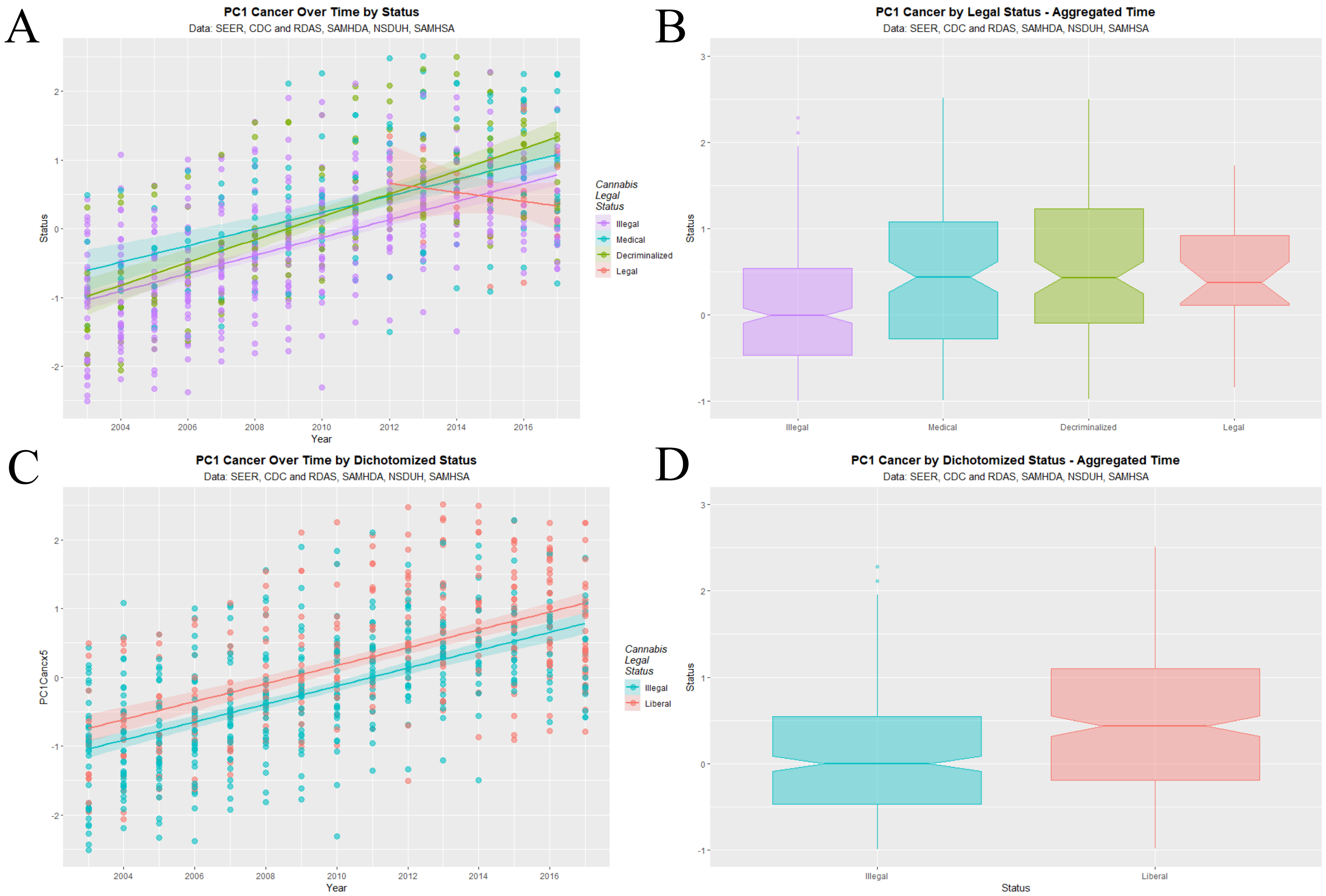

Supplementary Figure 18.: Effect of Cannabis legal status on PC1 for cancer data. (A) scatterplot over time for PC1 for cancer's for legal status. (B) Boxplot for PC1 for cancer incidence by legal status with aggregated time. (C) Scatterplot over time for PC1 for cancer incidence aggregated over time dichotomized as liberal v. illegal regimes. (D) Boxplot for PC1 for cancer incidence of dichotomized legal status over aggregated time dichotomized as in (C). (Created in R-Studio version 1.3.1093 using ggplot version 3.3.2).
